# When Flooding Is Not Catastrophic—Woven Gas Diffusion Electrodes Enable Stable CO_2_ Electrolysis

**DOI:** 10.1021/acsaem.2c02783

**Published:** 2022-12-08

**Authors:** Lorenz M. Baumgartner, Christel I. Koopman, Antoni Forner-Cuenca, David A. Vermaas

**Affiliations:** † Department of Chemical Engineering, Delft University of Technology, Van der Maasweg 9, 2629 HZDelft, Netherlands; ‡ Department of Chemical Engineering and Chemistry, Eindhoven University of Technology, Het Kranenveld 14, 5612 AZEindhoven, Netherlands

**Keywords:** CO_2_ reduction, electrochemistry, electrochemical engineering, gas diffusion electrode, scale-up

## Abstract

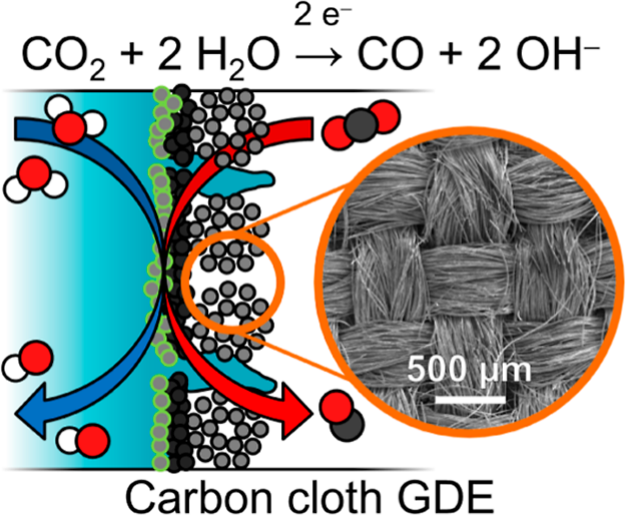

Electrochemical CO_2_ reduction has the potential to use excess renewable electricity to produce hydrocarbon chemicals and fuels. Gas diffusion electrodes (GDEs) allow overcoming the limitations of CO_2_ mass transfer but are sensitive to flooding from (hydrostatic) pressure differences, which inhibits upscaling. We investigate the effect of the flooding behavior on the CO_2_ reduction performance. Our study includes six commercial gas diffusion layer materials with different microstructures (carbon cloth and carbon paper) and thicknesses coated with a Ag catalyst and exposed to differential pressures corresponding to different flow regimes (gas breakthrough, flow-by, and liquid breakthrough). We show that physical electrowetting further limits the flow-by regime at commercially relevant current densities (≥200 mA cm^–2^), which reduces the Faradaic efficiency for CO (FE_CO_) for most carbon papers. However, the carbon cloth GDE maintains its high CO_2_ reduction performance despite being flooded with the electrolyte due to its bimodal pore structure. Exposed to pressure differences equivalent to 100 cm height, the carbon cloth is able to sustain an average FE_CO_ of 69% at 200 mA cm^–2^ even when the liquid continuously breaks through. CO_2_ electrolyzers with carbon cloth GDEs are therefore promising for scale-up because they enable high CO_2_ reduction efficiency while tolerating a broad range of flow regimes.

## 1 Introduction

Electrochemical CO_2_ reduction (CO_2_R) might be a key technology in our efforts to de-fossilize the chemical industry and transport sector with renewable electricity generated by wind or solar power.^1,2^ This process could convert CO_2_, which has been captured from point sources or directly from the atmosphere,^3−5^ to useful chemical intermediates. Depending on the catalyst, common target intermediates include CO (Ag),^6,7^ C_2_H_4_ (Cu),^8,9^ or HCOOH (Sn).^10,11^ Recently, the production of methanol and/or ethanol has been demonstrated with Cu_2_O/ZnO catalysts^12,13^ or metal–organic frameworks.^14−16^ These conversion products could then be further upgraded to produce liquid hydrocarbon fuels or plastics aiming for a CO_2_ neutral process.

Currently, a key challenge for the wide-scale adoption of CO_2_R is designing an electrolyzer that can operate at high Faradaic efficiency, high current density, and low cell voltage. The reactor also has to be scalable and operate stably for tens of thousands of hours. Liquid-fed electrolyzers suffer from CO_2_ mass-transfer limitations that lead to an increase in the undesired hydrogen evolution reaction (HER) at high current densities. To overcome this restriction, the field has introduced gas diffusion electrodes (GDEs), which allow the supply of CO_2_ directly from the gas phase to the electrocatalytic interfaces. This development step has allowed high Faradaic efficiency at industrially relevant current densities (≥200 mA cm^–2^).^17−19^

GDEs have been successfully integrated into two major types of gas-fed CO_2_ electrolyzers. In electrolyzers with a membrane electrode assembly (MEA), the cathode GDE is in direct contact with a membrane. The GDE exchanges ions with the anode and a flowing electrolyte, which are on the other side of the membrane.^20−22^ In electrolyzers with a flowing catholyte, the GDE is in direct contact with an electrolyte. This electrolyte layer adds additional ohmic losses but allows better control of the ionic environment at the reaction interface.^8,10,23−25^

In a typical GDE, gaseous reagents transfer from the gas channel through the carbon fiber substrate (CFS) and the microporous layer (MPL) before reaching the catalyst layer (CL).^26,27^ The CFS can have different microstructures (carbon paper, carbon cloth, and nonwoven) and is typically impregnated with polytetrafluoroethylene (PTFE) to provide wet-proofing. The MPL consists of carbon particles and PTFE. This layer plays an important role in controlling the intrusion of liquid into the gas diffusion layer (GDL)^28^ and improves the electrical contact with the CL. The CL consists of catalyst particles in an ionomer matrix and requires ionic contact with the adjacent membrane or electrolyte.^19,29^

Many studies have found that the flooding of the GDE with electrolyte is a major challenge for maintaining high selectivity for CO_2_R, especially at high current densities and a larger electrolyzer scale. When flooding occurs, the electrolyte infiltrates the pore network, which reduces the effective diffusivity of the GDE and ultimately results in the flooding of the porous structure.^30,31^ This phenomenon has been reported for both MEA-based and catholyte-based reactor configurations.

When focusing on CO_2_ electrolyzers with a flowing catholyte, the GDE can flood if the differential pressure between the liquid and the gas phases, Δ*p* = *p*_L_ – *p*_G_, exceeds the interfacial forces of the pore network. Therefore, the flooding behavior depends on the differential pressure^32^ but also on the wetting properties and microstructure.^33^ The flooding behavior is made even more complex by electrowetting. This physical phenomenon reduces the hydrophobicity of a surface when an electrical potential is applied.^32,34,35^

**Figure 1:**
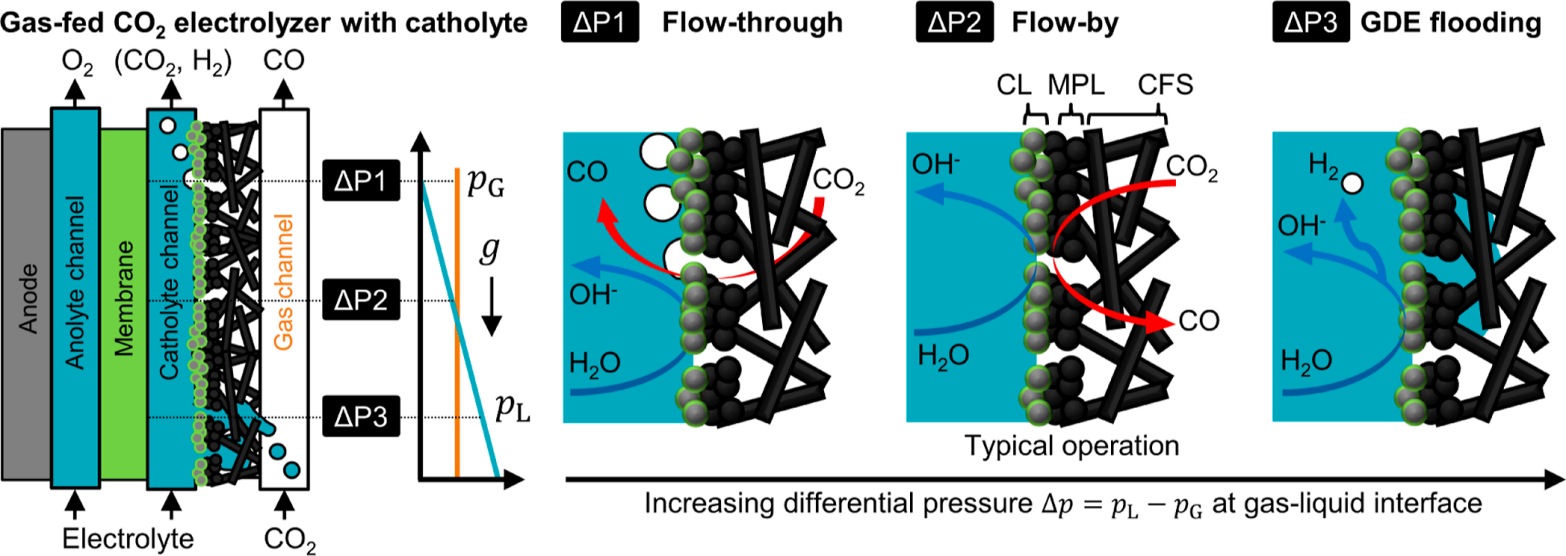
Flow regimes at the GDE of a gas-fed CO_2_ electrolyzer with flowing catholyte. Hydrostatic (and/or hydrodynamic) pressure gradients along the liquid channel can lead to a pressure imbalance at the gas–liquid interface. Flow-through regime (ΔP1): gas overpressure leads to the breakthrough of CO_2_ bubbles to the liquid phase. CO_2_R occurs on sections of the CL that have sufficient contact with the electrolyte. Flow-by regime (ΔP2): interfacial forces keep the GDL dry at low pressure differences between the gas and liquid phases. This ensures mass transfer of CO_2_ through the CFS and the MPL to the CL. GDE flooding regime (ΔP3): liquid overpressure leads to the flooding of the GDL and breakthrough of electrolyte into the gas channel. The flooding of pores can reduce the transfer of CO_2_ and favor the HER at the CL.

While the effect of pressure differences across the GDE on the CO_2_R performance has been receiving more attention recently,^32,36^ its importance for scale-up has received limited attention.^34^ At the same time, the scale-up of electrolyzers with a gas–liquid interface at the GDE inherently involves a non-uniform hydrostatic (and/or hydrodynamic) pressure balance.^37−39^ The difference in density between the gas and liquid phases leads to a variation in Δ*p*, which can change the local flow regime along the GDE (Figure 1). In the flow-through regime,^36^ gas breakthrough occurs because Δ*p* is lower than the capillary forces of the pore network (ΔP1). In the flow-by regime, no breakthrough occurs as the pressure of the gas and the liquid phase are balanced (ΔP2). In the GDE flooding regime, Δ*p* is sufficiently high to push the electrolyte into the pore network and liquid breakthrough can occur (ΔP3).

This raises the question of how the flow regime at the GDE actually impacts the performance of the CO_2_ electrolysis reaction. In this work, we study how the GDE structure and the operating conditions (cathode potential and differential pressure) affect the flooding behavior and performance of the gas-fed CO_2_ electrolyzer with a flowing catholyte. We measured the Faradaic efficiency for CO with an electrolysis setup that allowed the control of the differential pressure across the GDE. For the first time, we show the impact of electrowetting *in operando* at an industrially relevant current density (200 mA cm^–2^). For this purpose, we applied an Ag CL to a selection of GDL substrates featuring different CFS microstructures (paper and cloth) and GDE thicknesses (250–450 μm).

We found that the cathode potential and GDE microstructure have a strong impact at those differential pressures different GDE flow regimes occur. Our results suggest that large-scale gas-fed CO_2_ electrolyzers with flowing catholyte do not have to be operated with a flow-by regime over the entire electrode area. GDEs with a suitable structure allow robust CO_2_ reduction despite flooding and electrolyte breakthrough as long as the gas channel can be drained at a sufficient rate. This insight offers a promising route to scale up CO_2_ electrolyzers using the currently available GDL materials.

## 2 Experimental Methods

We prepared GDEs from a selection of commercial GDL substrates. We examined the gas–liquid flow regimes and electrochemical performance in a gas-fed CO_2_ electrolysis cell with flowing catholyte. More detailed descriptions of the experimental procedures are available in the Supporting Information.

The selection of commercial GDL materials was obtained from Fuel Cell Store (USA). We studied the effect of CFS thickness with a series of Toray carbon papers (TGP-H-060, 090, 120). We investigated the effect of pore size distribution (PSD) by comparing the Toray papers with SGL carbon papers (22BB, 39BC) and a carbon cloth (ELAT LT1400W). The CFS of all substrates had been wet proofed with PTFE by the manufacturer. The microstructure was visualized by scanning electron microscopy (SEM).

The GDEs were prepared by coating the GDL substrate with an automated airbrush coating system (Figure S1). The deposited CL had a target catalyst loading of 1 mg Ag cm^–2^ and a target composition of 80 wt % Ag nanoparticles and 20 wt % Nafion 521 ionomer. After cutting the GDL to size, we mounted the sample to the heating plate (130 °C) of the system and covered it with a 3 × 3 cm stencil. We prepared the ink by adding 33 mg of Ag nanopowder (20–40 nm, 99.9%, Alfa Aesar), 2.1 mL of deionized water, 2.1 mL of isopropyl alcohol, and 180 μL of Nafion D-521 dispersion (5 wt %, Alfa Aesar) in a glass vial. After homogenizing the ink for 30 min in a sonication bath, we used the airbrush and a 2D motorized stage to spray it evenly onto the MPL side of the GDL substrate.

**Figure 2:**
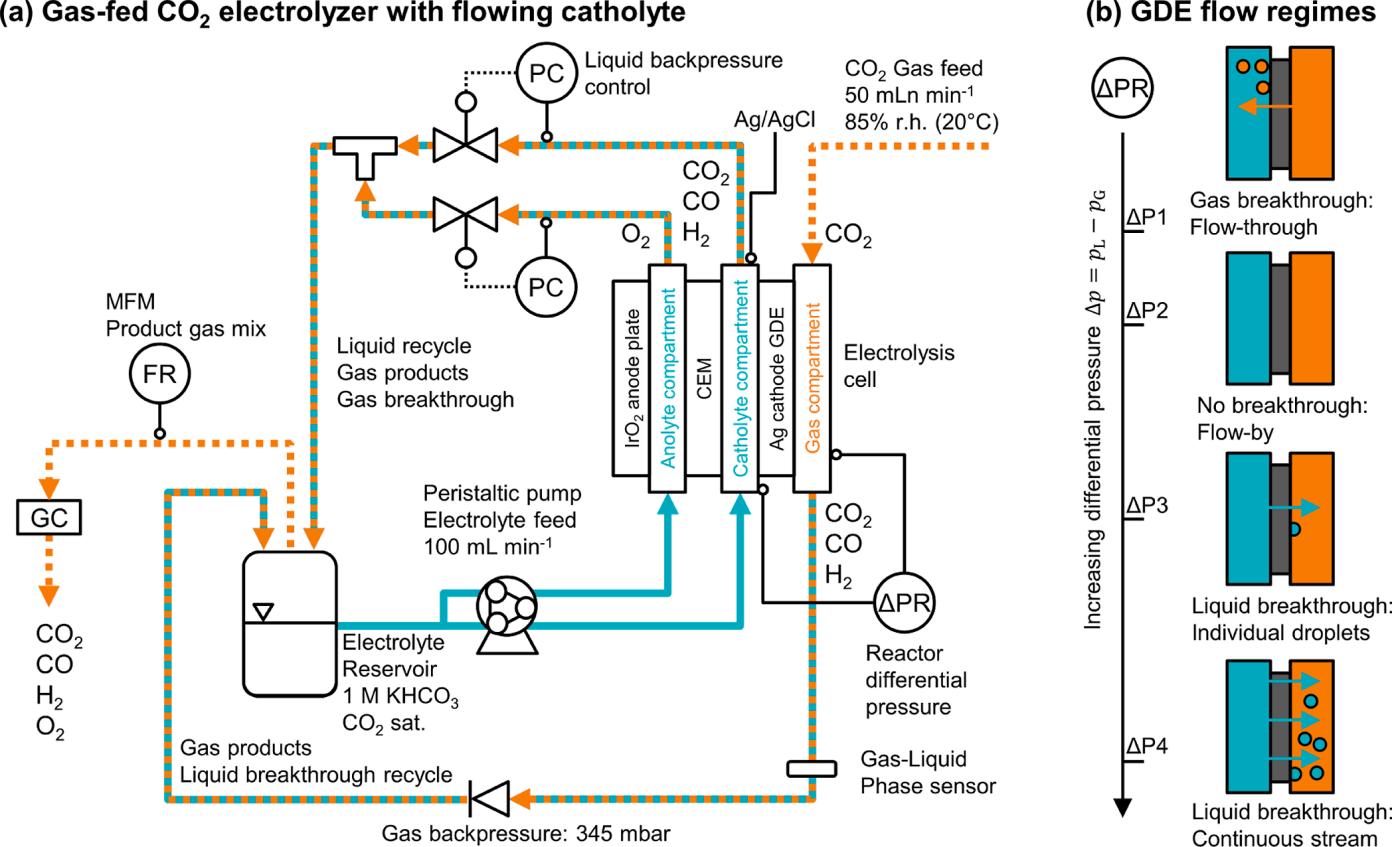
(a) Process flow diagram of the CO_2_ electrolysis setup with differential pressure, Δ*p*, control. The anolyte and catholyte compartments were separated with a cation exchange membrane. The backpressure of both electrolyte streams was controlled (PC) before the two liquid streams were combined and recirculated. We directly measured Δ*p* between the catholyte and gas compartment (ΔPR). The cathode potential was recorded with a Ag/AgCl reference electrode. The Faradaic efficiency was determined by recording the flow rate with a MFM and analyzing the gas composition by GC. (b) Gas–liquid flow regimes observed at the GDE. ΔP1: start of gas breakthrough (flow-through) and transition to separated flow, ΔP2: no gas or liquid breakthrough (flow-by)—liquid and gas phase were separated, ΔP3: individual liquid droplets form on the gas side and run down GDE, and ΔP4: continuous liquid stream through GDE.

We studied the effect of the different GDE flow regimes on the CO_2_ reduction performance with the electrolysis setup shown in Figure 2a. The humidified CO_2_ feed was passed through the gas compartment of the flow cell. We used a gas–liquid phase sensor to estimate the volumetric fraction of the electrolyte present in the product gas stream at the outlet of the flow cell. The back pressure was set by a check valve with a cracking pressure of 345 mbar. The peristaltic pump supplied the two liquid compartments with saturated 1 M KHCO_3._ We recorded the differential pressure between the gas and the catholyte compartment. Two electronic valves controlled the liquid back pressure. We collected the product gas mixture in the head space of the electrolyte reservoir and recorded its flow rate with a mass flow meter (MFM). The composition was determined with gas chromatography (GC).

After inserting a dry GDE sample into the electrolysis cell, we increased the liquid backpressure until liquid breakthrough occurred. Through this initial flooding, we aimed to eliminate the effect of the residual liquid saturation, which causes differences between the first and subsequent flooding-drainage cycles (see Section 7.2 in the Supporting Information).^30^ We repeated the following steps for each current density (0, 10, 100, and 200 mA cm^–2^): the liquid back pressure was reduced until gas breakthrough was observed, after which the galvanostatic control of the potentiostat was started.

We increased the liquid back pressure to control the differential pressure between the gas and liquid phases. This allowed us to establish the four characteristic flow regimes at the gas–liquid interface (Figure 2b): (ΔP1) start of gas flow-by (slight gas breakthrough), (ΔP2) flow-by (no breakthrough), (ΔP3) individual droplets breaking through, and (ΔP4) a continuous liquid stream breaking through. After the system was equilibrated for 6 min at each flow regime, we carried out three GC injections to determine the Faradaic efficiency for CO. Then, the CO_2_ electrolysis procedure was repeated at the next current density. An overview of the experimental sequence is shown in Figure S11.

## 3 Results and Discussion

We investigated the interfacial phenomena at the gas–liquid interface and the CO_2_ reduction performance for a selection of commercial GDL substrates. Supplementary results and the numerical values of all plotted data are included in the Supporting Information.

### 3.1 Physical Characterization of GDEs

**Figure 3:**
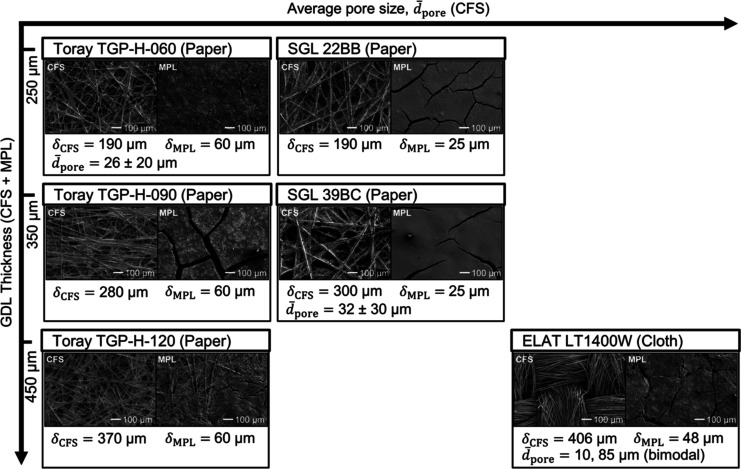
Microstructure and property data of commercial GDE substrates: SEM images at 100x magnification. The thickness of the CFS, δ_CFS_, was obtained from the manufacturer and supplier data sheets. The layer thicknesses of ELAT LT1400W were provided by the FuelCellsEtc GDL Comparison table. The average diameter of the CFS pores, , was obtained from Parikh et al. for the carbon paper and nonwoven GDLs.^41^ The bimodal PSD of the carbon cloth is based on an ELAT Nuvant cloth.^42^ Toray papers: the CFS was wet-proofed with 8–9 wt % PTFE; the MPL with 33–35 wt % PTFE. SGL papers: the CFS was wet proofed with 5 wt % PTFE; the MPL with 23 wt %. The ELAT carbon cloth was also impregnated, but the PTFE content was unavailable.

The microstructures of the different GDL materials are illustrated by SEM images (Figure 3). We arranged the materials in the order of the CFS thickness, δ_CFS_, and the average CFS pore size,  Carbon papers are made of carbon fiber fragments that are held together by organic binders. This random lacing makes them spatially uniform in the in-plane direction of the material.^40^ The Toray papers (TGP-H-060, 090, and 120) have a CFS with a finer, unimodal PSD with small amounts of binder. The SGL papers (22BB and 39BC), in contrast, have a broader, unimodal PSD and a large amount of binder, which gives the CFS a coarser structure. The finer structure and narrower PSD of the Toray papers is also reflected in the smaller value of  and its smaller standard deviation (Toray: 26 ± 20 μm vs SGL: 32 ± 30 μm).^41^ The CFS of the LT1400W carbon cloth (ELAT) is woven from carbon fiber bundles without a binder. This structure makes them anisotropic in the in-plane direction^40^ and leads to a bimodal PSD, which has large pores between the fiber bundles  and smaller pores  between the individual carbon fibers.^42^ The  of the CFS, in conclusion, increased in the following order: Toray paper < SGL paper < cloth.^42^

Although the MPL of our materials vary in thickness (Figure 3), we assume that the flooding properties will be mostly determined by the CFS because the large cracks in the MPL offers little flooding resistance.^33^ The CFS and MPL of our substrates were impregnated with different amounts of PTFE (Figure 3). Literature studies show that the effect of PTFE content on wettability levels off after exceeding a certain loading threshold (e.g., 10 wt %).^43,44^ We measured very similar static contact angles for all GDLs,^33^ which suggests that differences in PTFE content should have little effect on the wettability.

### 3.2 Pressure for Flow-By Regime Depends on the Microstructure and Cathode Potential

**Figure 4:**
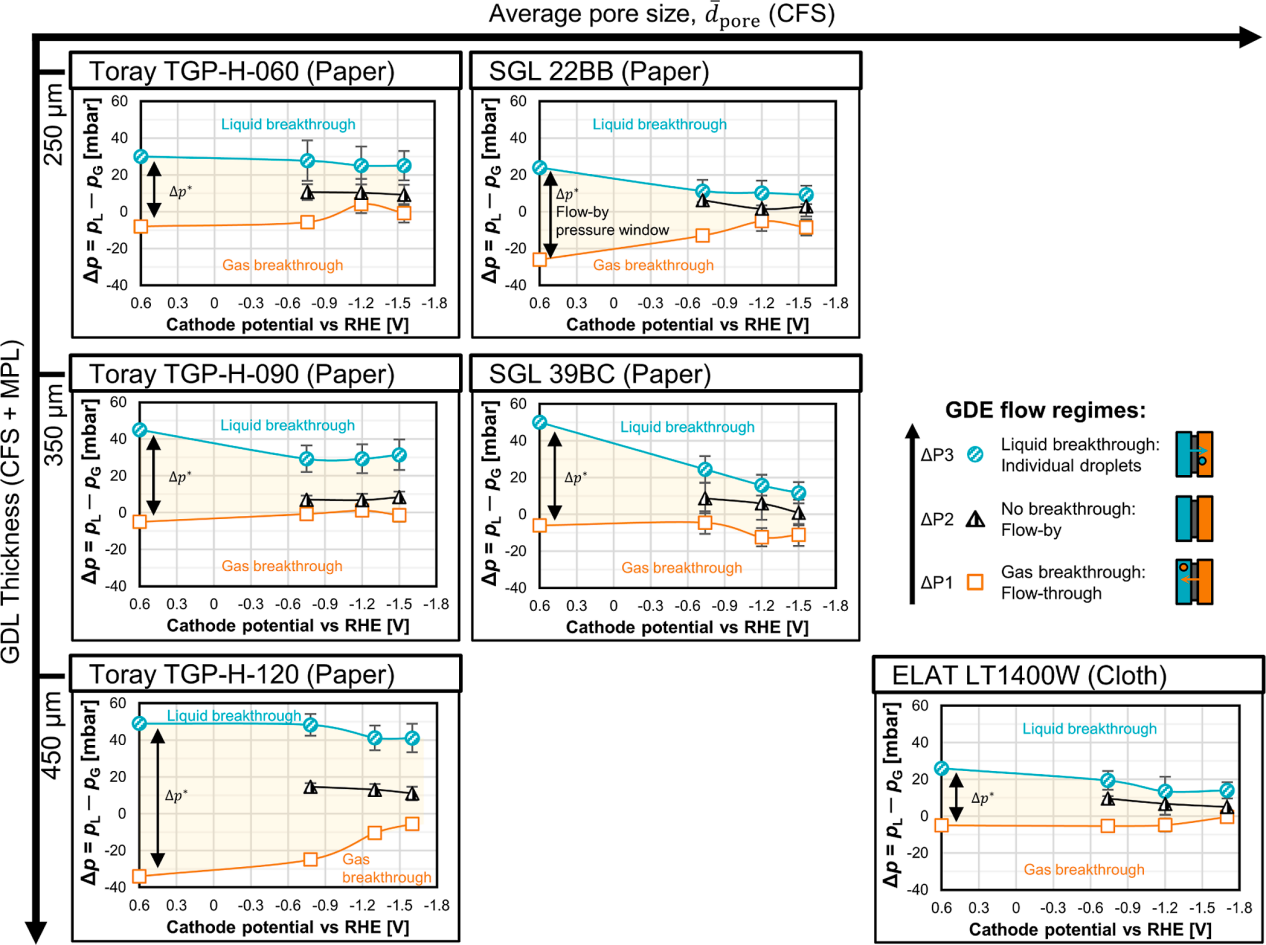
Development of the flow-by pressure window, Δp*, as a function of GDE microstructure and the cathode potential. The cathode potential is plotted relative to the RHE and was compensated for the *iR*-drop. The shaded yellow area between the curves for ΔP1 and ΔP3 indicates Δp*. In the vertical direction, from bottom to top, the markers represent the observed GDE flow regimes: the square markers (□) indicate ΔP1, the pressure points at which gas breakthrough starts; the shaded triangle markers (△) indicate ΔP2, a series of pressure points in the flow-by regime; and the shaded round markers (○) indicate ΔP3, the pressure points at which electrolyte breakthrough starts. In the horizontal direction, from left to right, the series of markers correspond to the current densities of 0, −10, −100, and −200 mA cm^–2^. The error bars indicate the standard deviation of the Δ*p* fluctuation during the experiment. Smaller error bars are covered by the markers.

From a previous work, we know that breakthrough of gas or liquids depends on the differential pressure Δ*p* = *p*_L_ – *p*_G_.^33^ However, those measurements were carried out at open circuit potential. When applying a potential to the cathode, it appears that the transition between the GDE flow regimes also depends on the cathode potential (Figure 4). We define the pressure zone, in which no gas or liquid breakthrough occurs, as the flow-by pressure window, Δp*. It is indicated by the yellow shaded area.

Δp* is the widest when no current is applied, and the GDE is at open circuit potential, which is at approximately 0.6 V versus reversible hydrogen electrode (RHE). Generally, we would expect a larger CFS thickness and a narrower PSD to widen Δp*. The impact of structural effects on Δp* has been discussed in more detail in a previous work.^33^ For instance, TGP-H-120 has a larger CFS thickness than TGP-H-060 (370 vs 190 μm), which results in a higher Δp*: 83 versus 38 mbar (Figure 4). The effect of  seems to depend on the type of GDL (paper or cloth) and/or the CFS thickness. While being similar in thickness, the carbon paper TGP-H-120 has a smaller  than the carbon cloth LT1400W. This structural difference results in a higher Δp*: 83 versus 31 mbar. However,  does not affect Δp* significantly for the thinner Toray and SGL carbon papers (≤350 μm). Note that the data in Figure 4 constitute a worst case scenario for Δp* because they were recorded with wet GDEs, which exhibit a narrower Δp* than initially dry GDEs (Figure S13).

The value of Δp* decreases for all materials if the cathode potential is reduced below the open circuit potential (Figure 4). For example, the Δp* of SGL 39BC drops by more than 50% from 56 mbar at open circuit potential (≙0 mA cm^–2^) to 23 mbar at −1.5 V versus RHE (≙–200 mA cm^–2^). The reduction in liquid breakthrough pressure is probably caused by reversible, physical electrowetting because we observed no permanent reduction in the static contact angle of the CFS after the CO_2_ electrolysis.^33^ The phenomenon of electrowetting reduces the hydrophobicity of an electrically charged surface because solvated ions are drawn into the electrical double layer.^35,45^ It is remarkable, however, that we observed such a strong change in the breakthrough pressure. For example, according to the Young–Laplace equation,^46^ we would expect the contact angle of water in a pore with a radius of 10 μm to drop from 110 to 100° to explain a reduction in capillary pressure from 50 to 25 mbar. To achieve such a drop in contact angle on a flat, dielectric PTFE surface, however, has been shown to require a potential of at least 50 V.^47^

A recent study of electrowetting on silver-based GDEs demonstrated that significant wettability changes occur at much lower potential differences (1 V) on bare metallic surfaces.^48^ We therefore hypothesize that the electrowetting on our GDEs does not predominantly take place on the insulating PTFE but instead takes place on uncoated carbon surfaces. The electrowetting behavior is also influenced by the heterogeneity and the rough surfaces inside the GDE’s pores. Hydrophobic, insulating PTFE is dispersed on conductive carbon surfaces (e.g., carbon fibers). At open circuit potential, the electrolyte likely rests on top of the rough, dispersed PTFE in a Cassie–Baxter wetting state. As the electrical potential is changed, the electrolyte probably transitions to a Wenzel wetting state^35,49^ by spreading along the uncoated, conductive carbon domains. The understanding of electrowetting in carbon-based GDEs could be greatly improved by future studies with operando synchrotron imaging.^48,50,51^

We would like to distinguish the reversible, physical electrowetting effect from irreversible (electro-)chemical degradation, which can decrease the contact angle of susceptible GDL materials such as the Freudenberg H23C6 permanently.^33^ This GDL substrate undergoes electrochemical degradation at cathode potentials below −0.65 V versus RHE.^52^ We hypothesize that the H23C6’s carbon fibers are graphitized to a lower degree during manufacturing to make them more flexible, but this also reduces their chemical stability.^33^

We also observed the gas breakthrough threshold to shift to a more positive Δ*p* for the samples SGL 22BB, TGP-H-060, and TGP-H-120 (Figure 4). This is a curious phenomenon because we would expect the gas breakthrough pressure to remain constant as long as the pores remain hydrophobic and gas filled. Starting at cathode potentials of −1.2 V versus RHE, bubbles form at the liquid side of the CL. These bubbles might also displace the electrolyte from previously wetted pores and thereby reduce the resistance against gas breakthrough (more positive Δ*p*).

The potential-dependent contraction of Δp* shows that it would be even more difficult to operate CO_2_ electrolyzers in the flow-by mode at a large scale when a significant current is applied. As the detrimental electrowetting effect reduces the resistance against electrolyte flooding, the cell height has to be limited to prevent electrolyte breakthrough due to hydrostatic pressure differences. Of the materials we studied (Figure 4), TGP-H-120 supports the widest flow-by pressure window of 47 mbar at −1.7 V versus RHE (≙–200 mA cm^–2^). This pressure window would correspond to a cell height of about 48 cm, which is relatively modest in comparison with the height of commercial cells for alkaline electrolysis (100–200 cm)^53^ or chlor-alkali electrolysis with an oxygen depolarized cathode (100–150 cm).^54,55^

### 3.3 Liquid Breakthrough Flow Rate Depends Primarily on Microstructure

Having established that breakthrough seems inevitable for large-scale GDEs operating between a liquid and a gas phase, the rate of breakthrough becomes a relevant metric. From a practical perspective, liquid breakthrough will be preferred over gas breakthrough, as the gas bubbles would cause additional ohmic resistances in the liquid compartment.^36^ Therefore, we used a gas–liquid phase sensor at the gas compartment outlet to estimate the liquid breakthrough flow rate, *F*_L_, when a current is applied (see Section 7.3 of the Supporting Information). The effect of differential pressure, Δ*p*, and cathode potential on *F*_L_ is show in Figure S16.

Materials with a thicker CFS and smaller average CFS pore size, , require a higher Δ*p* to allow the same liquid breakthrough flow rate, *F*_L_. The thinner TGP-H-060, for instance, requires an average Δ*p* of 46 mbar to force an *F*_L_ of 6.3 mL min^–1^ cm^–2^ (Figure S16). The thicker TGP-H-090, in contrast, requires 58 mbar to achieve the same flow rate. This phenomenon can be explained by the higher hydrodynamic pressure drop imposed by the longer flow path through the thicker GDL. Similarly, the pressure drop is also increased by smaller (44) which is well illustrated by the comparison of the ELAT cloth with the TGP-H-120 paper. The larger pores of the cloth permit an average *F*_L_ of 5.1 mL min^–1^ cm^–2^ at 26 mbar, while the narrower pores of the carbon paper permit 3.6 mL min^–1^ cm^–2^ at 53 mbar (Figure S16).

The electrowetting effect does not seem to have a strong influence on the permeability, as *F*_L_ does not vary significantly as a function of the cathode potential for all GDE materials (Figure S16). This limited effect of electrowetting could mean that the increasing wettability does not establish many new percolation pathways but branches out the flooded pore volume inside of the network. From a hydrodynamic perspective, we can expect new pathways to only contribute marginally to the overall percolation flow because they have a smaller pore diameter than the already flooded pores. According to the Hagen–Poiseuille equation, the flow rate through a pore scales with the fourth power of the diameter . The relationship between the overall *F*_L_ and Δ*p* is, therefore, mostly determined by the large pores in the percolation flow path, which are already flooded at higher (less negative) cathode potentials. Advanced imaging techniques, such as X-ray computed tomography,^56−58^ would greatly enhance the understanding of these complex two-phase flow dynamics inside a GDE under operating conditions.

### 3.4 Faradaic Efficiency for CO Depends on the Microstructure and GDE Flow Regime

**Figure 5:**
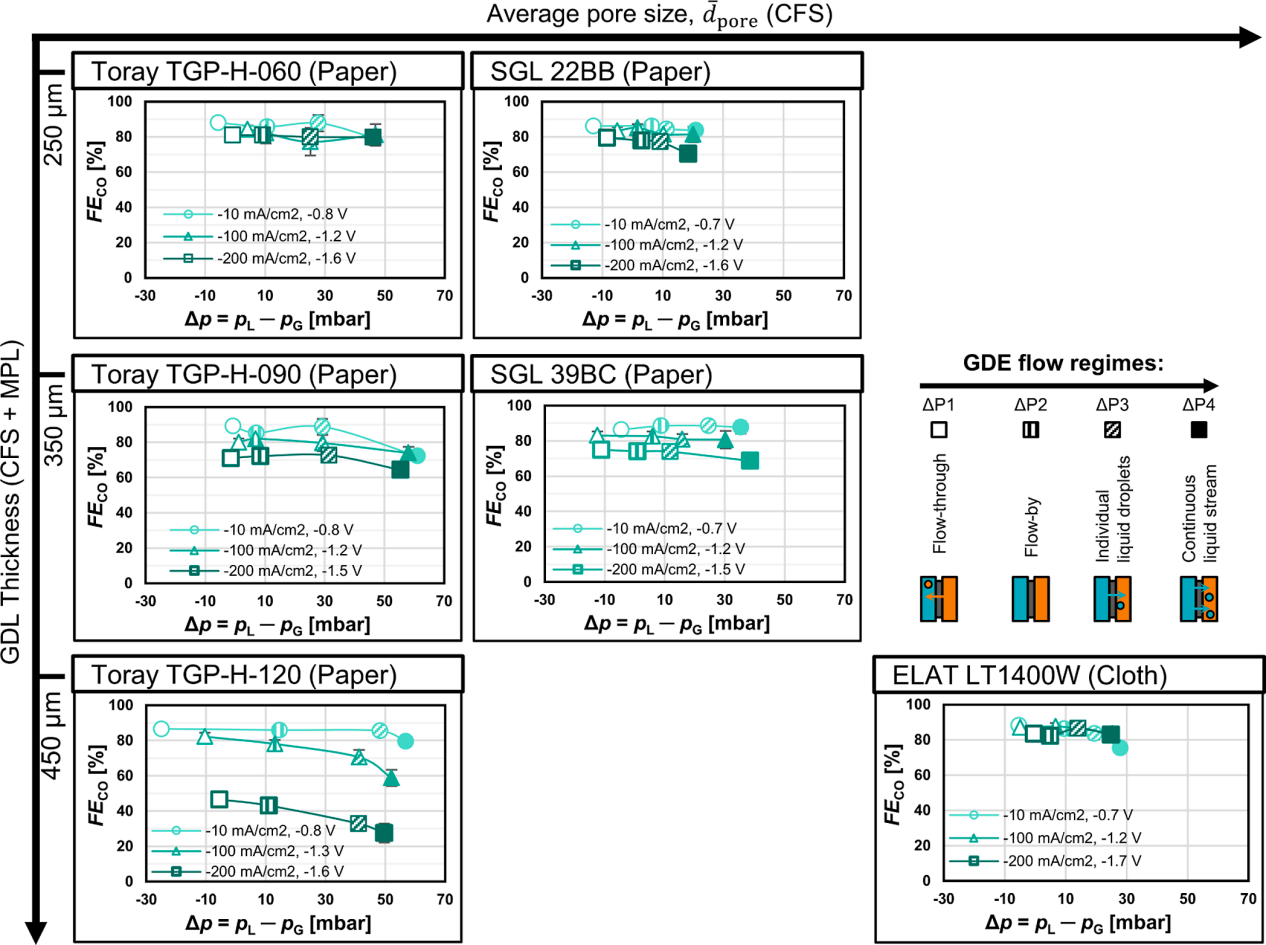
Faradaic efficiency for CO, *FE*_CO_, as a function of differential pressure, Δ*p*. The data series correspond to the current densities −10, −100, and −200 mA cm^–2^ from lighter to darker colors. The corresponding cathode potential against the RHE is given in the legend of each diagram. The marker filling indicates the GDE flow regime. The *y*-axis error represents the standard error for three consecutive GC injections. Smaller error bars are covered by the marker. The *x*-axis error bars were omitted here to make the representation of the other data clearer. These error bars are identical with the *y*-axis error bars in Figure 4.

To assess the impact of gas and liquid breakthrough on the Faradaic efficiency for CO, *FE*_CO_, we experimentally tested *FE*_CO_ for each GDE at different Δ*p*, thereby inducing flow regimes of gas breakthrough, flow-by, or liquid breakthrough (Figure 5). For each current density curve, the different marker fillings indicate the flow regime with increasing differential pressure Δ*p*. Empty marker (ΔP1): start of gas flow-through, first shaded marker (ΔP2): flow-by, second shaded marker (ΔP3): individual liquid droplets breaking through, and filled marker (ΔP4): continuous liquid stream breaking through. We listed the cathode potential next to the legend for each current density curve because this potential showed little dependence on Δ*p* for most materials (Figure S17). The ELAT carbon cloth seems to be an exception to this because it deformed mechanically (discussion in Section 7.4 of Supporting Information). We tested the stability of the GDEs by repeating the current density step of –100 mA cm^–2^ for two substrates (Figure S18).

The highest *FE*_CO_ is achieved by materials with thinner CFS and/or larger , which allow higher transport rates of CO_2_ at higher current densities (Figure 5). If the supply of electrons surpasses the diffusional flux of CO_2_, the excess current is then shifted to the undesired HER. For example, TGP-H-060 has a thinner CFS than TGP-H-120 (190 vs 370 μm) and thus exhibits a significantly higher *FE*_CO_ (81 vs 46%) at –200 mA cm^–2^ and ΔP1 (Figure 5). Similarly, the broader PSD of the LT1400W cloth in comparison with the TGP-H-120 paper results in a superior *FE*_CO_ (84 vs 46%) at –200 mA cm^–2^ and ΔP1 (Figure 5). The higher FE_CO_ achieved with thinner and/or coarser CFS structure (larger ) was already known for the stable pressure window^33^ and is now also confirmed for breakthrough regimes. We note that the apparent effects of CFS thickness and CFS pore structure have to be treated with caution when comparing materials from Toray with materials from SGL or ELAT because there are also differences in the MPL structures (Figure 3).

The CO_2_R performance generally drops with increasing Δ*p* (Figure 5). For instance, the FE_CO_ at –200 mA cm^–2^ for SGL 39BC drops from 75 to 69% when Δ*p* is increased from ΔP1 to ΔP4. The liquid saturation in the pore network of the GDE increases with Δ*p*, which leads to a lower effective diffusivity for gaseous reactants.^30,31^ This diminishes the mass transfer of CO_2_ to the CL and reduces the rate of CO_2_R in favor of the unwanted HER. The magnitude of this effect, however, depends strongly on the GDE structure.

**Figure 6:**
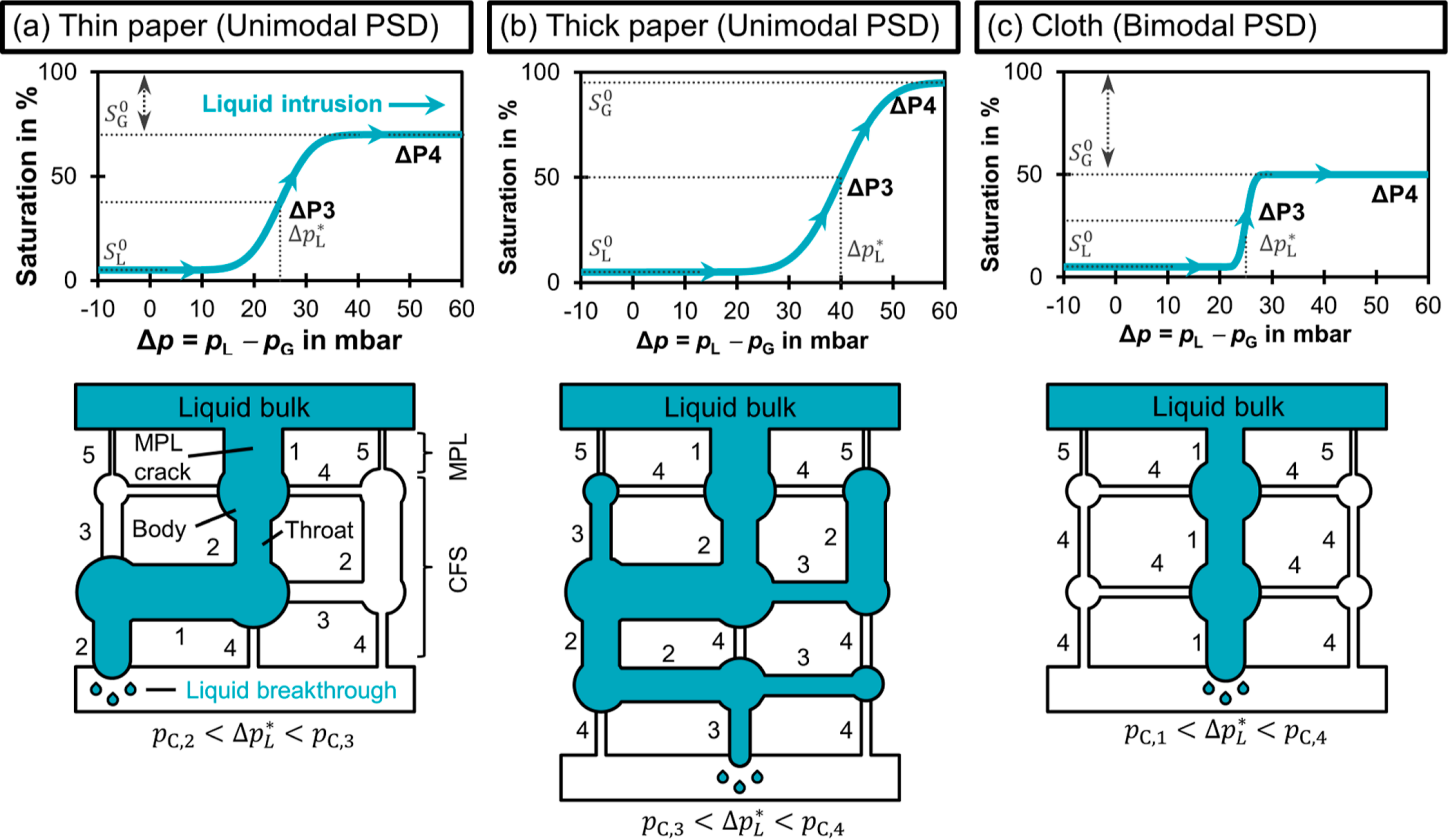
Saturation behavior of different CFS structures. The hypothetical saturation curves show how the liquid intrusion changes the saturation level. The curves start at their residual liquid saturation, *S*_L_^0^, as the GDEs were pre-wetted. As Δ*p* is increased, the saturation ultimately reaches the full effective saturation, at which the residual gas saturation, *S*_G_^0^, remains unflooded.^30,59^ The schematic pore networks^33,58,60^ explain the difference in saturation at the percolation threshold (ΔP3). The spatial connectivity of the pores determines the percolation flow path and the liquid breakthrough pressure, Δ*p*_L_*. The relative order of capillary pressures is *p*_C,1_ < *p*_C,2_ < *p*_C,3_ < *p*_C,4_ < *p*_C,5_. Cracks allow the liquid to bypass the pores with high capillary pressure (*p*_C,5_) of the MPL. (a) Thin paper: the intruding liquid has to overcome *p*_C,2_ and takes a relatively straight path through the material. (b) Thick paper: the additional layer increases Δ*p*_L_^*^ by adding a pore with *p*_C,3_ to the flow path. This allows the liquid to branch out more and reach a higher saturation. (c) Cloth: the liquid follows a direct flow path along pores with the *p*_C,1_. No branching occurs because Δ*p* is too low to flood adjacent pores. This leads to high *S*_G_^0^.

The CO_2_R performance of thicker carbon papers falls as a consequence of electrolyte intrusion (Figure 5). The thick TGP-H-120, for example, shows a drop in FE_CO_ from 46 to 27% at –200 mA cm^–2^ when Δ*p* is increased from ΔP1 to ΔP4. In contrast, the thin TGP-H-060 shows an insignificant drop in *FE*_CO_ from 81 to 80% for the same conditions. We can explain the different effects for thin and thick carbon papers with qualitative saturation curves^30^ and schematic pore network models^33,58,60^ (Figure 6). The connectivity of the pore bodies (circles) and throats (rectangles) determines the flow path that the intruding liquid follows. Each throat resists flooding up to its capillary pressure, *p*_C,i_.

We hypothesize that a thin paper becomes less saturated because the intruding liquid is drained at a lower liquid breakthrough pressure or percolation threshold, Δ*p*_L_^*^ or ΔP3 (Figure 6a). This prevents the liquid from branching out extensively inside the pore network and lets the thin paper maintain a higher residual gas saturation, *S*_G_^0^, when Δ*p* is increased further.

A thick paper, in comparison, has a higher Δ*p*_L_^*^ because the longer flow path has a higher probability of including a throat with a high capillary pressure.^61^Figure 6b illustrates this effect with the additional layer of the thick paper, which adds a throat with *p*_C,3_ to the flow path. We think that the additional thickness leads to stronger branching out of the liquid in two ways. First, the higher Δ*p*_L_^*^ allows pores with higher *p*_C,i_ to be flooded. Second, the longer percolation flow path increases the probability of the liquid to be in contact with pores that can be flooded. Thus, there are less uninterrupted flow paths in the gas phase, which reduces the effective diffusivity and leads to a lower *FE*_CO_ with increasing Δ*p*.

The high CO_2_R performance of the LT1400W carbon cloth is only minimally affected by electrolyte intrusion (Figure 5). This can be seen by the insignificant reduction of *FE*_CO_ from 84 to 83% at –200 mA cm^–2^ when comparing ΔP1 to ΔP4. This behavior can be attributed to the bimodal PSD of the cloth which preferentially drains the electrolyte through the large pores between the fiber bundles^44^ and leaves the adjacent smaller pores available for gas diffusion (Figure 6c). We therefore hypothesize that the carbon cloth has the highest *S*_G_^0^ of the investigated materials, which allows high CO_2_ transport even if liquid breakthrough is occurring.

**Figure 7:**
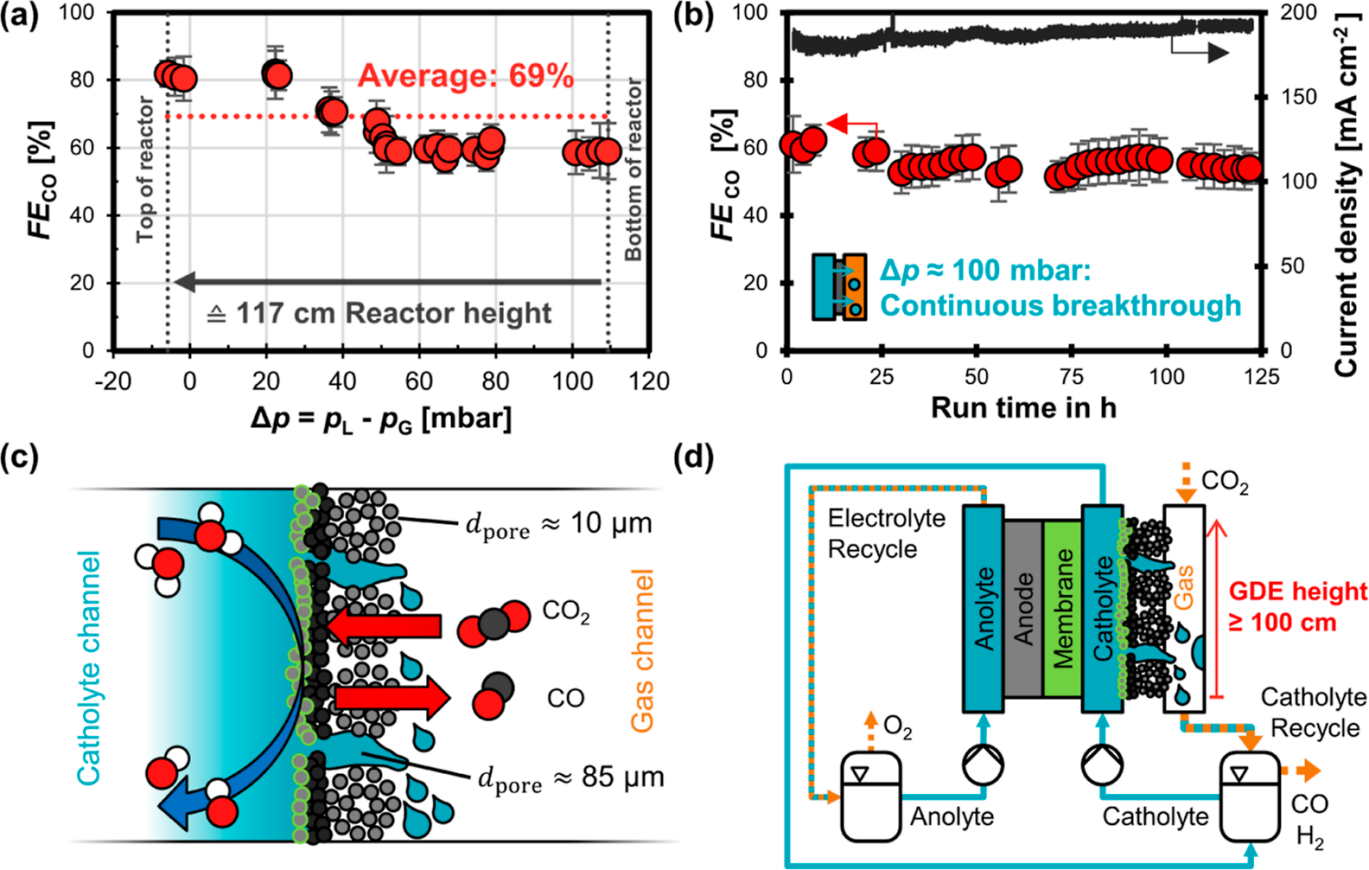
CO_2_R performance test of ELAT LT1400W carbon cloth GDE. (a) Faradaic efficiency for CO, *FE*_CO_, as a function of differential pressure, Δ*p*. The cell potential was constant at 10 V (potentiostat limit). The current density was between –180 and –200 mA cm^–2^. The average *FE*_CO_ was determined by integrating *FE*_CO_ numerically over the Δ*p* range. (b) Robust CO_2_ electrolysis despite continuous catholyte breakthrough: Δ*p* ranged from 80 to 120 mbar. The cell potential was constant at 10 V. The current density was between –180 and –193 mA cm^–2^. (c) Transport mechanisms inside flooded cloth GDE cloth: CO_2_ and gaseous products (CO, H_2_) can diffuse through the dry, small pores inside the fiber bundles. Liquid electrolyte can pass through the large pores between the fiber bundles. (d) Proposed scalable CO_2_ electrolyzer design: the cloth GDE allows robust CO_2_ conversion despite electrode breakthrough in lower sections of the cell. More detailed data on all experiments available in Section 8 of the Supporting Information.

The modest impact of breakthrough of gas or liquid on *FE*_CO_—at least for materials with bimodal pore structures or thin CFS—has a large practical meaning, as it suggests that the large-scale operation of CO_2_ electrolyzers is still possible at good selectivity, accepting breakthrough of gas or liquid. To further investigate this implication, we conducted a more stringent performance test to evaluate how well a GDE based on ELAT LT1400W would perform inside a cell with a height ≥ 100 cm. We varied Δ*p* from −6 to +109 mbar, which resulted in a mixed flow regime along the GDE and an average *FE*_CO_ of 69% (Figure 7a). More details on these experiments are available in Section 8 of the Supporting Information.

The cloth GDE allows robust CO_2_ reduction for at least 125 h at current densities close to –200 mA cm^–2^ despite experiencing continuous breakthrough due to a liquid overpressure Δ*p* of around 100 mbar. The Faradaic efficiency for CO remains between 55 and 60% (Figure 7b). The dips and slight decrease in *FE*_CO_ were caused by oxygen crossover (after stopping the purge gas) and interruptions in the control software, while the flooding does not seem to change the Faradaic efficiency significantly over time (Section 8 of the Supporting Information).

We hypothesize that the robust CO_2_ reduction is enabled by the bimodal pore structure of the cloth, which separates the transport pathways of the gas and electrolyte phase (Figure 7c). Electrolyte breakthrough must occur through cracks in the MPL and large pores between the fiber bundles of the cloth (*d*_pore_ ≈ 85 μm).^42^ The smaller pores within the fiber bundles (*d*_pore_ ≈ 10 μm) remained gas filled and allowed the CL to exchange CO_2_ and CO with the gas channel (Figure 7c). Using the capillary pressure equation provided by Wood et al.,^62^ we can estimate that Δ*p* would need to exceed 138 mbar before these small pores are also filled with electrolyte.

Based on the promising performance of the cloth GDE, we believe that GDEs with a bimodal PSD are able to maintain sufficient gas transport for CO_2_ electrolysis at high current densities even for continuous liquid breakthrough. Compared to operating at lower overpressure, the Faradaic efficiency is only slightly compromised (Figure 7a). We propose a CO_2_ electrolyzer design, which should be scalable to an electrode height of at least 100 cm (Figure 7d). The percolated catholyte is collected and separated from the product gas stream inside the catholyte reservoir.

Compared to MEA-based CO_2_ electrolyzers with anion exchange membranes,^21,63^ the use of a catholyte layer in our proposed design would act as a buffer between the membrane and the catalyst. This would allow the utilization of, for example, a bipolar membrane, which could reduce CO_2_ crossover^10,63,64^ and allow the deployment of a non-precious anode made from nickel.^10,25,65^ Although the catholyte channel introduces additional ohmic resistance, it allows better control of the local reaction environment at high current densities^8,25^ in comparison to MEA electrolyzers, in which the water management at the membrane^66^ and the cathode^67^ or salt formation in the gas channel^68,69^ can hinder performance. From a practical perspective, the reactor (Figure 7d) has to be fed with a sufficiently high electrolyte flow rate to ensure that it does not run dry. Liquid breakthrough rinses the GDE^34^ and the gas channel, which limits salt deposition from carbonate scaling.

## 4 Conclusions

We have studied how structural parameters (CFS structure, CFS thickness, and CFS pore size) and process parameters (differential pressure and cathode potential) influence the scalability of gas-fed CO_2_ electrolyzers with flowing catholyte.

The scale-up of an electrolyzer operating in a flow-by regime is not viable with the currently available commercial GDL materials. The relatively low capillary pressure and electrowetting make it difficult to keep the fluid phases separated at industrially relevant current densities (≥–200 mA cm^–2^). A thick carbon paper with a small average CFS pore size (Toray TGP-H-120) achieved the widest flow-by pressure window of 47 mbar, which corresponds to a relatively modest electrode height of 48 cm. The same structure, however, leads to poor diffusivity in the GDL, which limits FE_CO_ to less than 46%.

Instead, we propose the scale-up of an electrolyzer with a carbon cloth GDE, which can tolerate GDE flooding and electrolyte breakthrough. We found that carbon cloth (ELAT LT1400W) allowed the highest FE_CO_ of 84% at –200 mA cm^–2^. The bimodal PSD allows this GDE to maintain a high effective diffusivity at higher liquid overpressures. The intruding electrolyte preferentially floods the large pores between the fiber bundles and is drained before it can flood the smaller pores inside the bundles. This ensures that a significant share of the GDL pores remain available for gas diffusion despite electrolyte flooding. We demonstrated that this material allows stable CO production with *FE*_CO_ ≥ 55% over at least 125 h despite high liquid overpressures of 100 mbar. This promising electrolyzer design would therefore enable a cell height of at least 100 cm and operate at an estimated average *FE*_CO_ of 69% at –200 mA cm^–2^.

## Supporting Information Available

Qualitative PSDs, detailed GDE fabrication description, technical description of the electrolysis cell and setup, detailed description of CO_2_ electrolysis experiment, liquid breakthrough measurement with the gas–liquid phase sensor, post-electrolysis characterization, and detailed data for CO_2_ electrolysis performance test with carbon cloth (PDF)
Numerical values for all results and figures and all recorded process parameters for CO_2_ electrolysis experiments (XLSX)
