# Supplementary material for: When Flooding Is Not Catastrophic—Woven Gas Diffusion Electrodes Enable Stable CO2 Electrolysis
Source: ACS Appl Energy Mater. 2022 Dec 8;5(12):15125–35. doi: 10.1021/acsaem.2c02783 (PMC9795489; doi:10.1021/acsaem.2c02783)
Supplement: Supplementary file 1 — ae2c02783_si_001.pdf [file ae2c02783_si_001.pdf]

# SUPPORTING INFORMATION

## When Flooding is not Catastrophic – Woven Gas Diffusion Electrodes enable stable $CO_2$ electrolysis

Lorenz M. Baumgartner,[a] Christel I. Koopman,[a] Antoni Forner-Cuenca,[b] David A. Vermaas*,[a]

[a]*Department of Chemical Engineering, Delft University of Technology, Van der Maasweg 9, 2629 HZ Delft, Netherlands*

[b]*Department of Chemical Engineering and Chemistry, Eindhoven University of Technology, Het Kranenveld 14, 5612 AZ Eindhoven, Netherlands*

*Email: D.A.Vermaas@tudelft.nl*

**Supporting Information Content:**

- 32 Pages
- 13 Tables
- 24 Figures

# Contents

1. General information ........ 2

2. GDE preparation ........ 2

3. GDL substrates: Qualitative comparison of pore size distributions ........ 3

4. Microstructure inspection with Scanning Electron Microscopy (SEM) ........ 4

5. Assembly of 3-compartment $CO_2$ electrolysis cell ........ 4

6. Engineering of the $CO_2$ electrolysis setup ........ 7

7. $CO_2$ electrolysis with varying current density and differential pressure ........ 12

7.1 Overview of experimental sequence for GDE testing ........ 12

7.2 Flow-by pressure window of GDL, dry GDE, and wet GDE ........ 13

7.3 Stability during $CO_2$ electrolysis with varying differential pressures ........ 19

7.4 Liquid breakthrough as a function of differential pressure and current density ........ 15

7.5 Cathode potential as a function of differential pressure and current density ........ 18

8. $CO_2$ electrolysis performance test with carbon cloth ........ 21

8.1 Faradaic efficiency and cathode potential as a function of differential pressure ........ 21

8.2 Performance test with continuous liquid breakthrough ........ 23

8.3 Post-electrolysis characterization ........ 26

8.4 Capillary pressure calculation with Owens-Wendt model ........ 31

References ........ 31

# 1. General information

De-ionized water was used for all experiments. Detailed experimental results are available in the **Excel file** *SI_Baumgartner_Vermaas_2022b.xlsx* of the supporting information.

# 2. GDE preparation

We prepared each GDE by depositing the CL with a tailor-made automated airbrush coating system (**Figure S1**). We used the same GDE coating procedure and the same GDE samples as in our previous publication.[1]

### Sample preparation

We cut the GDL to size a size of 3.5 cm x 3 cm, dried it for 10 min at 120°C, and weighed it in an airtight container (Kartell 034600 Polypropylene Weighing Bottles – 50 mL, Fisher Scientific). We then covered the sample with a 3 cm x 3 cm PTFE mask and fixed it to the heating plate (130°C).

### Ink preparation

An example for the ink specifications is given in **Table S1**. The target catalyst loading was 1 mg Ag cm$^{-2}$. We selected this common catalyst loading to simplify the comparison with other studies.[2] The solid composition was 80 wt% Ag and 20 wt% Nafion 521 ionomer. The Nafion binder content of 20 wt% was selected to match the optimized content determined by Duarte *et al.*.[3] To prepare the catalyst ink, we added 33 mg of Ag nanopowder (Aerodynamic particle size (APS): 20–40 nm, 99.9%, Alfa Aesar), 2.1 mL of water, and 2.1 mL of isopropyl alcohol into a 10 mL glass vial. Then, we added 180 µL of Nafion D-521 dispersion (5 wt%, Alfa Aesar) to achieve an ink solid (Ag +Nafion) concentration of 1% g mL$^{-1}$. Note that we used an excess of ink to compensate for the loss of 30% ink during the deposition process. We homogenized the ink for 30 min in a sonication bath (USC500TH, VWR).

### Deposition process

The ink was evenly sprayed onto the MPL side of the GDL sample with a Paasche Airbrush Set TG3 (Airbrush Services Almere, Netherlands) mounted on a custom made 2D-motorized stage (**Figure S1**).

### Determination of catalyst loading

After the deposition process, we dried and weighed the coated sample for 10 min at 120°C to determine the mass of the CL. An overview of the catalyst loading per sample is given in **Table S2**.

**Table S1**: Example for catalyst ink specifications.

| Parameter | Unit | Value |
|---|---|---|
| Ag nanoparticle target loading | mg cm$^{-2}$ | 1.1 |
| Expected deposition efficiency | % | 30% |
| Spray-coated area | cm$^2$ | 9 |
| Required catalyst mass | mg | 33 |
| Weighed catalyst mass | mg | 33.4 |
| Nafion content in catalyst layer | wt% | 20 |
| Add Nafion solution (5 wt%) | mL | 0.180 |
| Ink solid concentration | w/v in g mL$^{-1}$ | 1% |
| Add $H_2O$:IPA (1:1) mixture | mL | 4.175 |

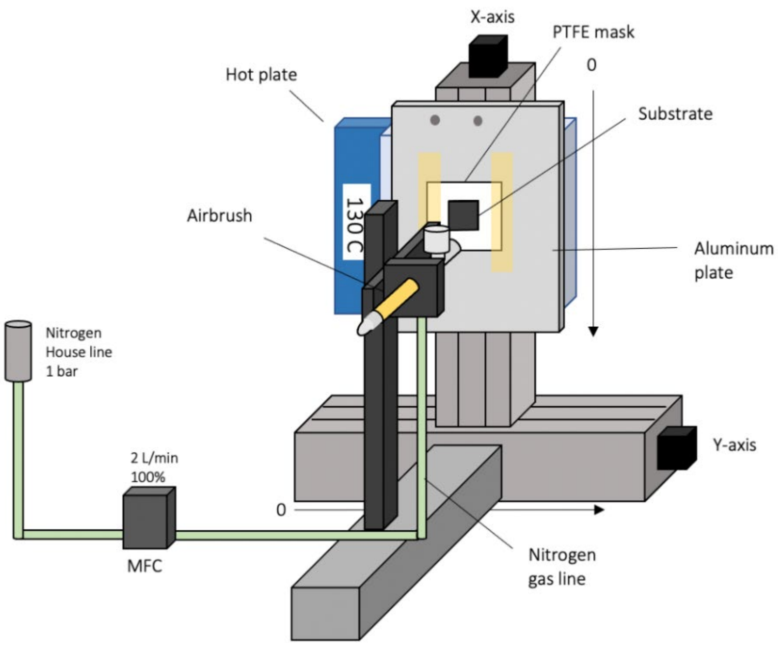


**Figure S1**: Catalyst layer deposition: Schematic of airbrush coating system.

**Table S2**: Summary of Ag catalyst loading in mg $cm^{-2}$ for our GDE samples.

| Material | TGP-H-060 | TGP-H-090 | TGP-H-120 | SGL 22BB | SGL 39BC | LT1400W |
|---|---|---|---|---|---|---|
| Ag loading | 1.16 | 1.08 | 1.05 | 1.29 | 1.40 | 1.20 |

## 3. GDL substrates: Qualitative comparison of pore size distributions

The studied GDLs exhibit the following trends from narrow to wide pore size distributions (PSD): Toray paper < SGL paper, Cloth (**Figure S2**). We ranked the carbon cloth according to its larger pores because these are relevant for the flooding resistance. While not all PSD data were available for this specific set of materials, we leverage measurements on substrates from the same set of materials which are expected to results in minor influences on the PSD (**Figure S2**). Forner-Cuenca *et al.* used materials without MPL and without PTFE wet-proofing. The Nuvant carbon cloth is of a different type than our LT1400W cloth.[4] Additional characterization data of all used GDE are available in the SI of our recent publication.[1]

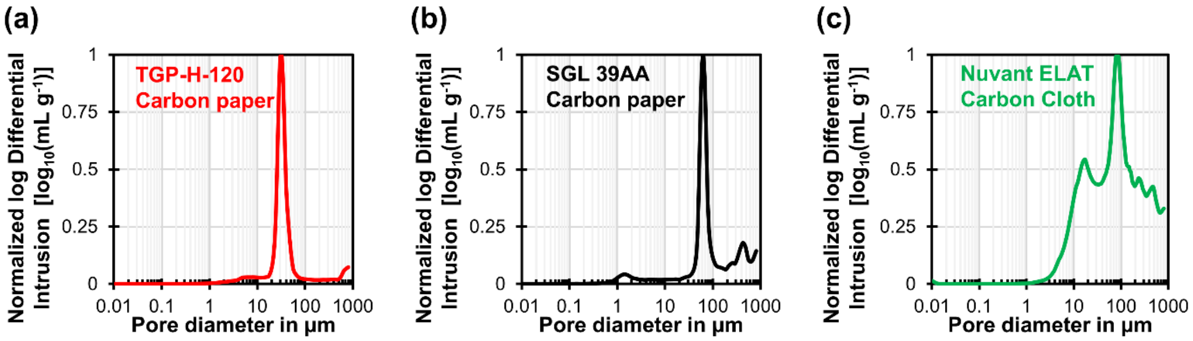


**Figure S2**: Qualitative comparison of the pore size distributions for the different carbon fiber substrate types. **(a)** ELAT carbon cloth **(b)** SGL carbon paper **(c)** Toray paper. Note that the substrates shown here differ from our study because they were not impregnated with PTFE and do not have a MPL. The data is based on mercury intrusion porosimetry measurements from Forner-Cuenca *et al.*.[4]

## 4. Microstructure inspection with Scanning Electron Microscopy (SEM)

The GDE microstructure was visualized with a JSM-6010LA SEM (JEOL, Japan). The instrument was equipped with a secondary electron imaging (SEI) detector for morphology and a backscattered electron composition (BEC) detector for elemental contrast imaging.

We characterized the CL with SEM images in our previous publication:[1] We estimate a thickness of 3.5 ± 0.2 µm. The CL consisted of a Nafion ionomer matrix with larger Ag agglomerates (200 – 1200 nm) embedded. The agglomerates were formed by smaller primary Ag particles (79 ± 17 nm).

## 5. Assembly of 3-compartment $CO_2$ electrolysis cell

The front view of the 3-compartment electrolysis cell is shown in **Figure S3**. The transparent body of the cell was machined out of poly methyl methacrylate (PMMA) by our workshop. This design allowed the observation of the flow regime at the GDE during operation. The cell was connected to tubing with fittings made of poly ether ether ketone (PEEK), which were supplied by the IDEX corporation (Illinois, USA). The PMMA parts were sealed against each other with 0.5 mm silicone gasket (Eriks, Netherlands). The cation exchange membrane (Selemion CMV ) was sandwiched between two gaskets. The differential pressure between the gas compartment and the catholyte compartment was measured directly inside the cell. For this purpose, the differential pressure meter was attached to the 1/16” outer diameter tubes, which were directly connected to the inside of the cell (**Figure S3**).

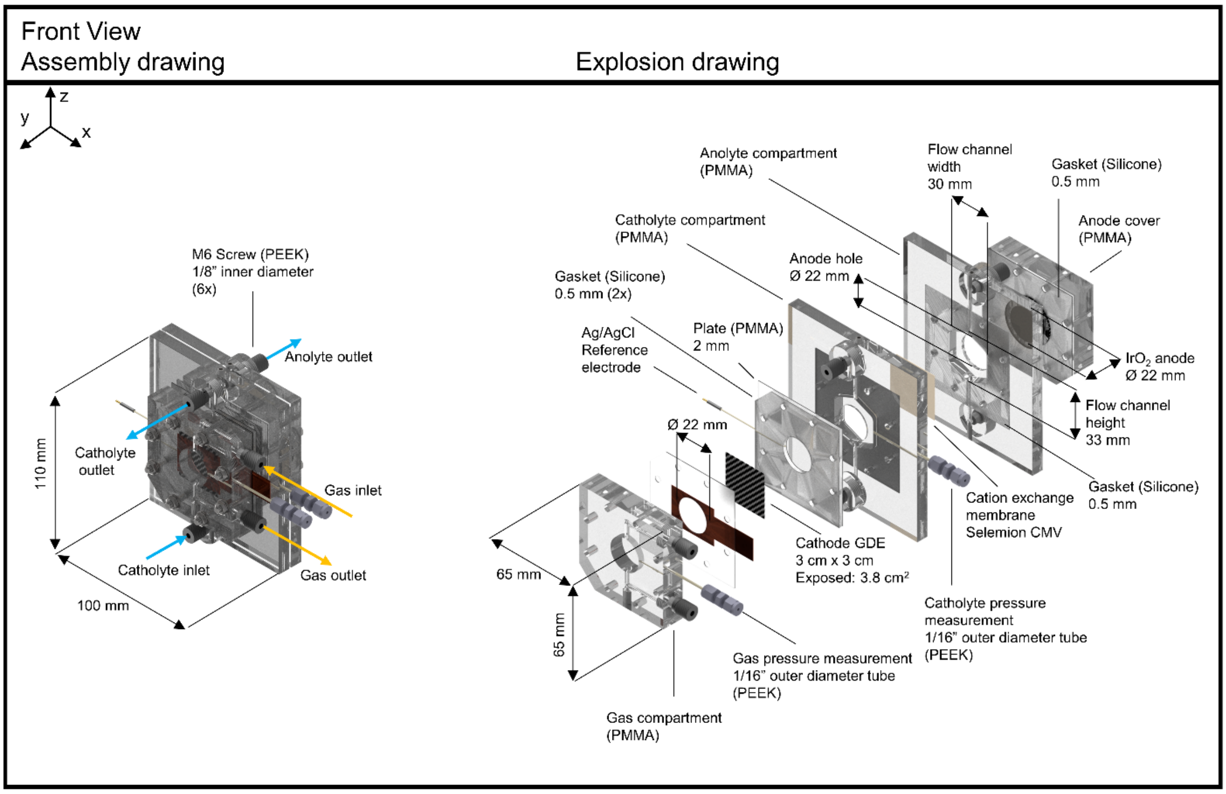


**Figure S3**: Front view of the 3-compartment electrolysis cell used to determine the flow-by pressure window pressure, $\Delta p_L^*$, and the Faradaic efficiency for CO, $FE_{CO}$, as a function of current density. PEEK stands for poly ether ether ketone. PMMA stands for poly methyl methacrylate.

The back view of the 3-compartment electrolysis cell is shown in **Figure S4**. The GDE was connected to the potentiostat with a copper tape (Conrad, Netherlands), which served as current collector. We integrated an Ag/AgCl

micro-reference electrode (Type: Leak free reference electrode; Supplier: Multi Channel Systems, Germany) into the center of the PMMA sheet that was next to the cathode GDE. The gap between cathode and tip of the reference electrode was 0.5 mm. The anode plate electrode was made of a titanium disk coated with an $IrO_2$ oxygen evolution catalyst (Magneto Special Anodes, Netherlands). We connected it to the potentiostat with a wire running to the back side of the disk through a hole in the anode cover.

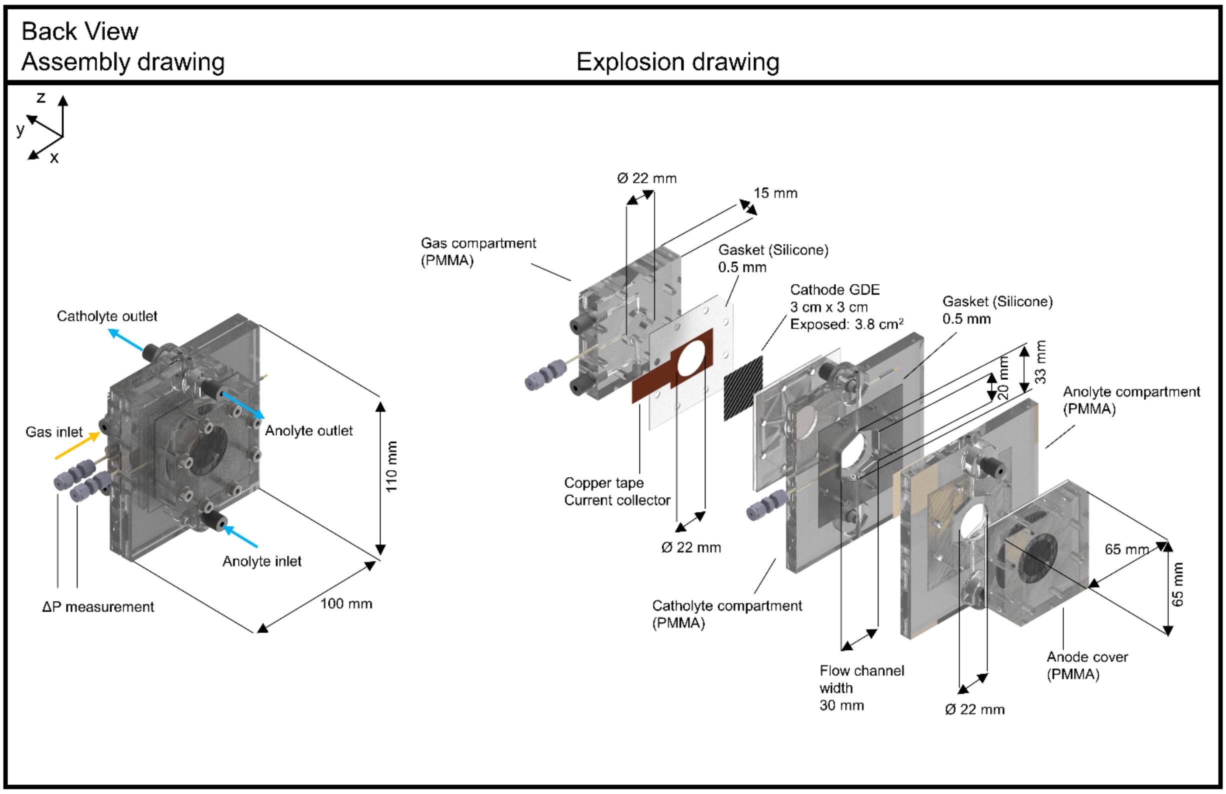


**Figure S4**: Back view of the 3-compartment electrolysis cell used to determine the flow-by pressure window pressure, $\Delta p_L^*$, and the Faradaic efficiency for CO, $FE_{CO}$, as a function of current density. PMMA stands for poly methyl methacrylate.

**Figure S5** shows the cross-section of the electrolysis flow cell. We choose and upward flow direction for the electrolytes to facilitate the removal of gas bubbles. The gas flows downward to remove any liquid from the gas compartment.

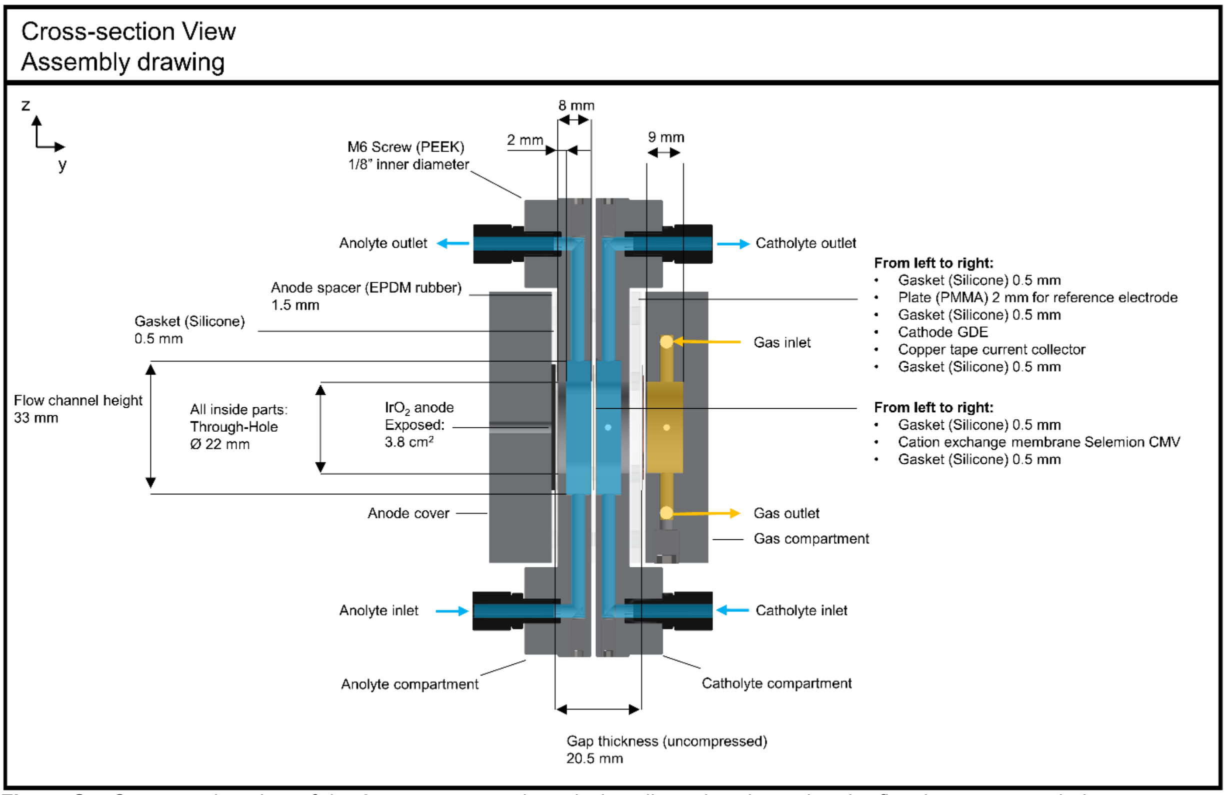


**Figure S5**: Cross-section view of the 3-compartment electrolysis cell used to determine the flow-by pressure window pressure, $\Delta p_{\mathrm{L}}^{*}$, at open circuit potential and the Faradaic efficiency for CO, $FE_{\mathrm{CO}}$, as a function of current density.

## 6. Engineering of the $CO_2$ electrolysis setup

The $CO_2$ reduction experiments were carried out with the electrolysis setup shown in **Figure S6** and **Figure S7**. We used Labview (Version 2018, National Instruments) to record online data of the various sensors and to control the pump and the electronic valves.

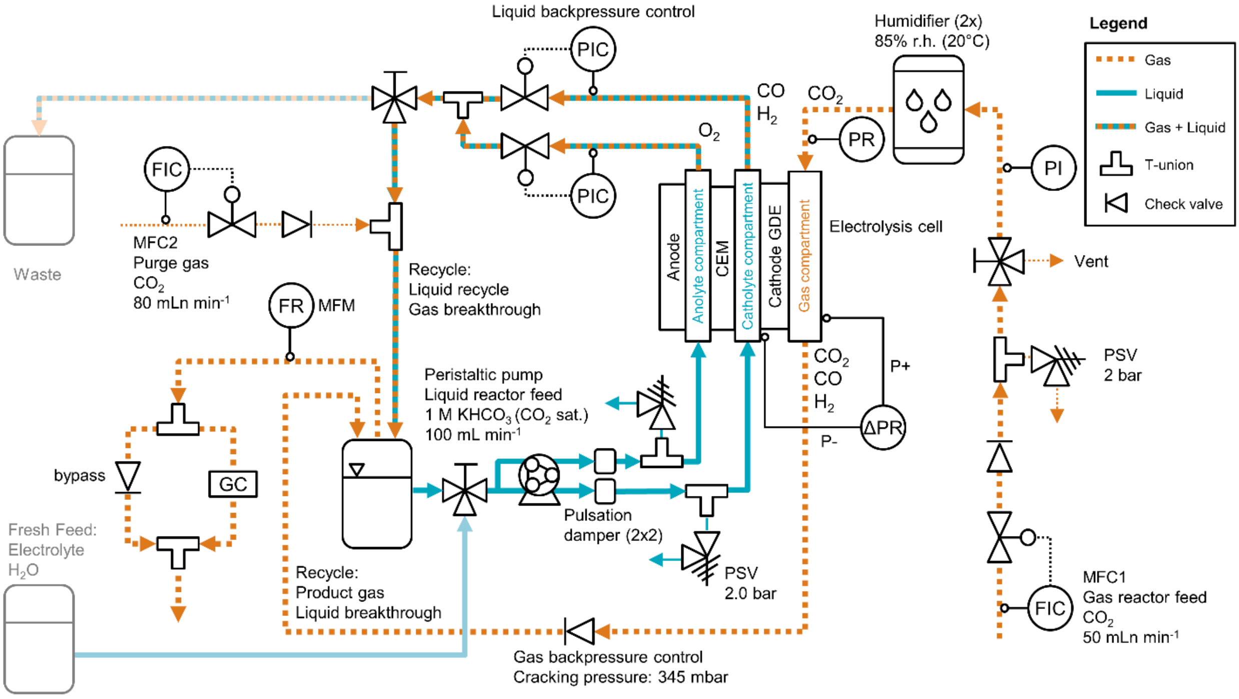


**Figure S6:** Extended process flow diagram for $CO_2$ electrolysis setup with differential pressure control. The gas flow rates were controlled with mass flow controllers (MFC). Check valves were used to prevent the backflow of liquid into the MFCs. Pressure safety valves (PSV) were installed in line to prevent the unexpected buildup of pressure. The gas feed pressure was measured with an analog pressure indicator (PI) and recorded after the humidifiers (PR). The differential pressure between the gas and the catholyte compartment was recorded with a differential pressure meter (ΔPR). The anolyte and catholyte compartment were separated with a cation exchange membrane (CEM). The backpressure of both electrolyte streams was controlled (PIC) before both streams were combined and recirculated. The product gases were collected from all process streams and combined in the head space of the electrolyte reservoir. Their flow rate was recorded (FR) with a mass flow meter (MFM) and the composition analyzed with a gas chromatography system (GC) to calculate the Faradaic efficiency. The containers for the fresh feed and waste are greyed out because they are not part of the main flow path.

### Gas feed flow path

The $CO_2$ feed gas was supplied from a $CO_2$ cylinder. The gas flow rate was controlled and measured with a mass flow controllers (MFC1) of the type F-201CV-500 from Bronkhorst (Netherlands). We passed the gas through two custom-made bubble columns (**Figure S8** and **Figure S9**) in series to humidify the feed with water vapor. The temperature and relative humidity of the gas feed was recorded after the humidification stage with a humidity sensor (Type: HC2A-S Hygroclip RV+T sensor; Supplier: Acin Instrumenten, Netherlands). The pressure of the gas feed was recorded with a Deltabar S pressure meter (Endress+Hauser, Switzerland). We used another Deltabar S to record the pressure difference between the gas compartment (positive terminal: P+) and the liquid compartment (negative terminal: P-). The backpressure of the gas outlet was set by a SS-CHS2-5 check valve (Swagelok, Netherlands) with a nominal cracking pressure of 345 mbar.

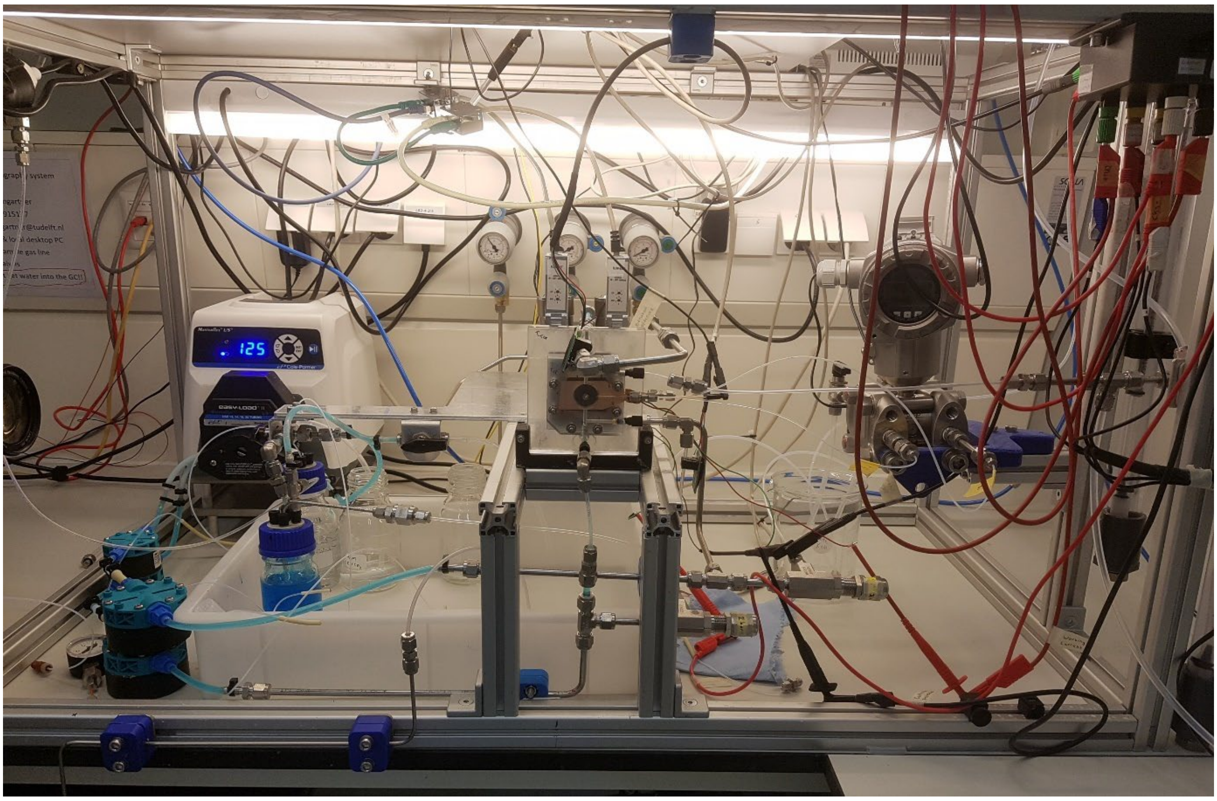

**Figure S7**: Picture of $CO_2$ electrolysis setup. The peristaltic pump is located on the left. The 3-compartment flow cell is in the center of the image.

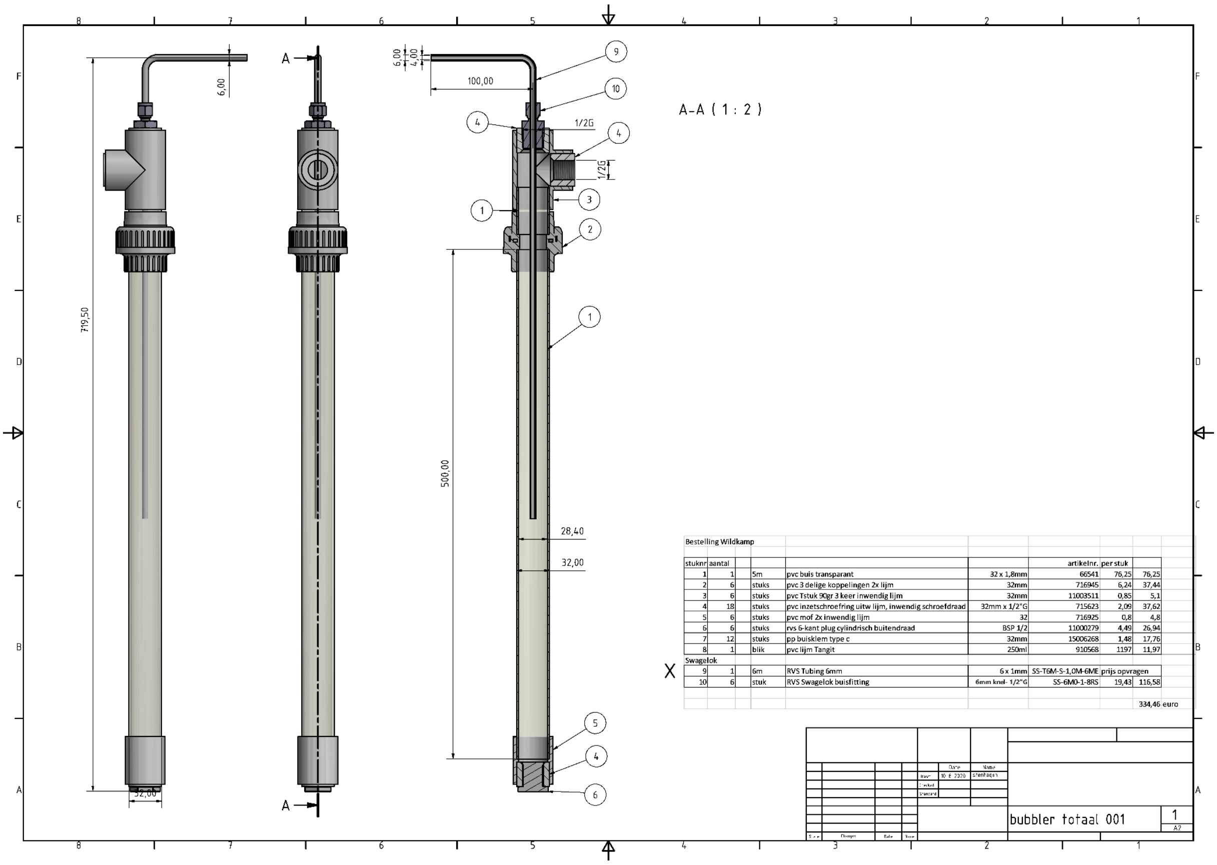


**Figure S8**: Technical drawing of the humidifier column. The units are in mm.

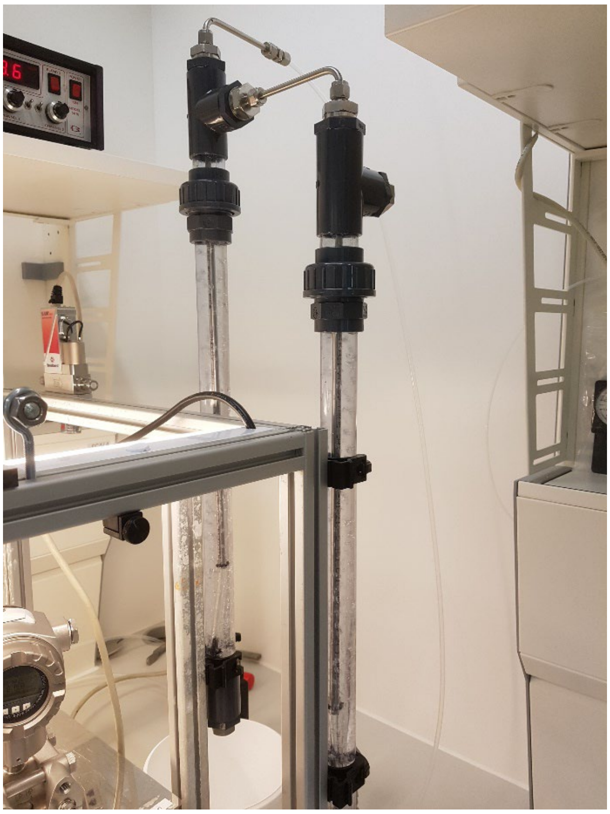

**Figure S9**: Two custom-made humidifier columns made from PVC pipes were used to humidify the $CO_2$ feed to 85 % relative humidity (r.h.) at 20°C.

**Electrolyte flow path**

The 1 M $KHCO_3$ electrolyte saturated with $CO_2$ was prepared by diluting concentrated KOH (50 wt%, analytical grade, Alfa Aesar) to 1 M KOH. The $CO_2$ was bubbled through the solution until the pH value was stable. The bulk pH of the electrolyte was measured prior to the experiments and is listed in the accompanying **Excel file**. The liquid lines and reactor were flushed before every experimental run. The electrolyte reservoir and liquid lines were filled with 60 mL fresh electrolyte. We used a peristaltic pump (Type: Masterflex L/S peristaltic pump; Supplier: Cole Parmer) to recirculate the electrolyte through the reactor and the liquid lines with a flow rate of 100 mL $min^{-1}$ for the catholyte channel and for the anolyte channel each. Two pulsation dampers (Types: FPD 1.06, FPD 1.10; Supplier: KNF, Switzerland) reduced the pressure fluctuations caused by the pump. We controlled the liquid back pressure of both channels with electronic control valves (Type: P-502C-6K0R; Supplier: Bronkhorst, Netherlands). After the experimental run, we collected a sample of the electrolyte to measure the formic acid content with HPLC. In addition to CO and $H_2$, formic acid has also been reported to form on Ag catalysts in small amounts.[5]

**Product gas flow path**

Unreacted $CO_2$ and product gases left the reactor through the gas outlet and entered the head space of the electrolyte reservoir. Product gases forming on the catholyte side (CO, $H_2$) and the anode side ($O_2$) were carried out of the reactor by the electrolyte stream. After the two electrolyte streams were recombined, we added a $CO_2$ purge gas stream to facilitate the transfer of product gases into the gas phase. The $CO_2$ purge gas stream further ensured that the electrolyte remained saturated with $CO_2$ during the experimental run. All the product gases were collected in the headspace of the electrolyte reservoir and passed through a mass flow meter (MFM) to record the flow rate (Type: F-111B-500; Supplier: Bronkhorst, Netherlands). The gas composition was analyzed with a gas chromatography system (Type: Compact GC 4.0; Supplier: Interscience, Netherlands).

**Calculation of Faradaic efficiency**

The Faradaic efficiency of gas species $FE_i$ ($H_2$, CO) was calculated with the recorded current, $I$, Faraday's constant, $F$, the stoichiometric number of electrons exchanged, $z_i$ ($z_i = 2$ for $H_2$ and CO), the corrected MFM gas flux, $\dot{N}_{\mathrm{MFM}}$, and the gas concentration, $C_i$, using (S1).

$$FE_i = \frac{z_i \cdot F \cdot C_i \cdot \dot{N}_{\mathrm{MFM}}}{I} \qquad \text{(S1)}$$

The simple gas conversion factors provided by the supplier of the MFM are listed in **Table S3**. We did not detect the components $CH_4$ or $C_2H_4$ in any of our product gas samples. Therefore, $CO_2$ is the only component that differs significantly from a conversion factor of 1 in our product mixture. This allows us to simplify the calculation of the mixture conversion factor $K_{\mathrm{mix}}$.

**Table S3**: Single component gas conversion factors $K_{\mathrm{i}}$ for 20°C and 1 atm provided by Bronkhorst General Manual Digital Instruments.

| Component | $K_{\mathrm{i}}$ |
|---|---|
| **$H_2$** | 1.01 |
| **$N_2$** | 1.00 |
| **$O_2$** | 0.98 |
| **$CO_2$** | 0.74 |
| **CO** | 1.00 |
| **$CH_4$** | 0.76 |
| **$C_2H_4$** | 0.60 |

We assume that the components $N_2$, $H_2$, $O_2$, are equivalent in their conversion factor to CO. We then developed a simple linear model using the Fluidat flow calculation tool (Bronkhorst, Netherlands). This tool allows to calculate $K_{\mathrm{mix}}$, which converts the recorded gas flow, $\dot{N}_{\mathrm{MFM,nominal}}$, (MFM calibrated for 90 vol% $CO_2$, 5 vol% CO, 5 vol% $H_2$ at 10 bar (a) and 20°C) to the corrected gas flow (actual product mixture at 0.1 bar (g) and 20°C). The corrected MFM gas flux, $\dot{N}_{\mathrm{MFM}}$, is then calculated with (S2). Our model assumes the product gas mixture is a two component mixture made up of $CO_2$ and CO (**Figure S10**).

$$\dot{N}_{\mathrm{MFM}} = \dot{N}_{\mathrm{MFM,nominal}} \cdot K_{\mathrm{mix}} \qquad \text{(S2)}$$

The mixture conversion factor, $K_{\mathrm{mix}}$, is calculated with the regression formula (S3) determined in **Figure S10**.

$$K_{\mathrm{mix}} = 1.291 - 0.2764 \cdot \frac{C_{\mathrm{CO_2}}}{[\mathrm{vol\%}]} \qquad \text{(S3)}$$

We calculated the volumetric concentration of $CO_2$, $C_{\mathrm{CO_2}}$, in vol % with (S4).

$$C_{\mathrm{CO_2}} = 100\ \mathrm{vol\%} - C_{\mathrm{CO}} - C_{\mathrm{H_2}} - C_{\mathrm{O_2}} - C_{\mathrm{N_2}} \qquad \text{(S4)}$$

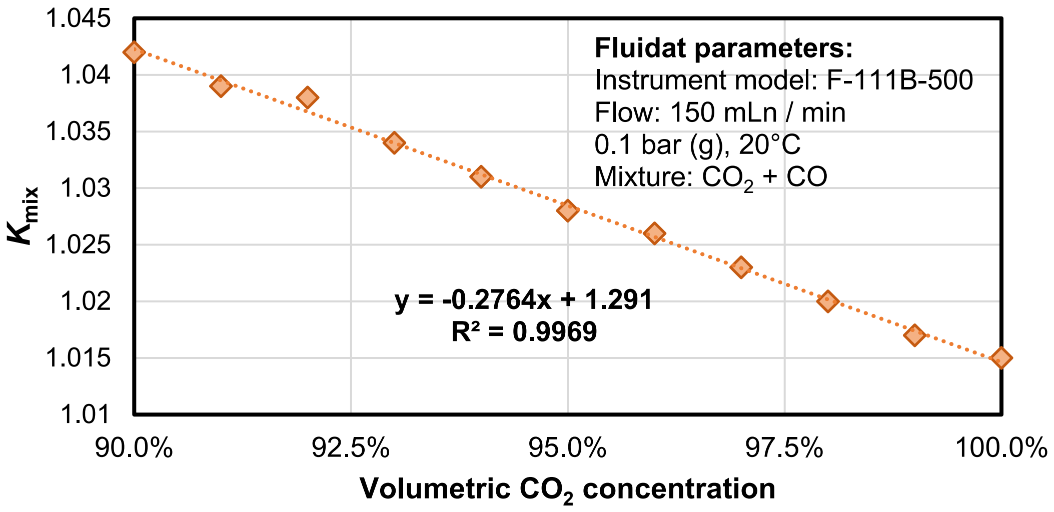


**Figure S10**: Determination of the mixture conversion factor, $K_{\mathrm{mix}}$: The linear regression model to calculate $K_{\mathrm{mix}}$ is based on data points calculated with the Fluidat flow calculation tool (Bronkhorst, Netherlands). The gas mixture consists of $CO_2$ and CO.

The Faradaic efficiency values of the product gases CO and $H_2$ are listed in the accompanying **Excel file**.

We can estimate the Faradaic efficiency of formic acid, $FE_{\mathrm{HCOOH}}$, with (S5). We collected a sample of the electrolyte after electrolysis and analyzed it with HPLC to determine the concentration of formic acid, $C_{\mathrm{HCOOH}}$, in mol $L^{-1}$. The total passed charge, $Q$, was recorded by the potentiostat. The electrolyte volume $V_{\mathrm{electrolyte}}$ was about 60 mL for each experiment.

$$FE_{\mathrm{HCOOH}} = \frac{2 \cdot F \cdot C_{\mathrm{HCOOH}} \cdot V_{\mathrm{electrolyte}}}{Q} \qquad \text{(S5)}$$

The detailed values for all GDL samples are listed in the accompanying **Excel file** for all experiments. We calculated a $FE_{\mathrm{HCOOH}}$ between 2% to 4% for our different materials samples. This roughly makes up the amount to complete the Faradaic efficiencies of $H_2$ and CO to 100%.

# 7. $CO_2$ electrolysis with varying current density and differential pressure

The following section contains more detailed descriptions of the methods and data processing for our $CO_2$ electrolysis experiments with varying current density and differential pressure for our six GDL materials. The sheet "**$CO_2$ electrolysis**" in the **accompanying Excel document** includes detailed values for all process parameters and resulting Faradaic efficiencies for $H_2$ and CO.

## 7.1 Overview of experimental sequence for GDE testing

The overview of the experimental sequence is presented in **Figure S11**. After installing the GDE in the electrolysis cell, we pre-flooded the GDE samples. During this procedure, we measured the flow-by pressure window, $\Delta p^*$, of the initially dry GDE by stepping the pressure up from ΔP1 to ΔP3 and recording the $\Delta p$ at which the GDE flow regime changed. Then, we measured the $\Delta p^*$ of the now wetted GDE by reducing the pressure back down to ΔP3 and recording when the liquid breakthrough ceased (ΔP3 of wet GDE) and when the gas breakthrough started (ΔP1).

Second, we carried out the $CO_2$ electrolysis run with the current densities 10, 100, and 200 mA cm$^{-2}$ at the different flow regimes ΔP1, ΔP2, ΔP3, and ΔP4 (**Figure S11**). Typically, the setting of each ΔP took less than 10 min. After the process parameters were set, we waited for 6 min so the system could equilibrate. We then carried out three GC injections.

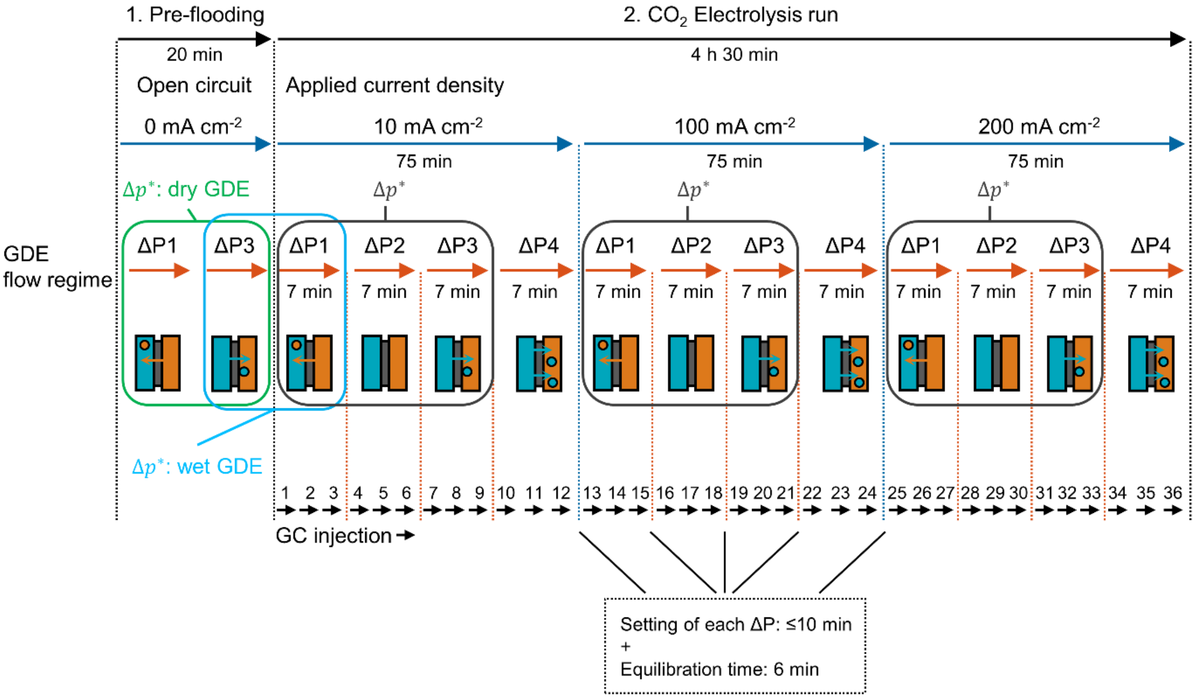


**Figure S11**: Overview of experimental sequence for GDE testing. **1. Pre-flooding** and measurement of the flow-by pressure windows, $\Delta p^*$, for the initially dry GDE and the pre-wetted GDE. **2. $CO_2$ electrolysis run** with the current densities 10, 100, and 200 mA cm$^{-2}$. During each current density step, we adjusted the liquid backpressure to set the four characteristic flow regimes at the GDE interface: (ΔP1) Transition from flow-through to flow-by, (ΔP2) Flow-by (no breakthrough), (ΔP3) Individual droplets breaking through, and (ΔP4): Continuous liquid stream breaking through. The setting of the pressure points took less than 10 min. For each parameter set of current density and GDE flow regime, we let the system equilibrate for 6 min before carrying out three GC injections.

## 7.2 Flow-by pressure window of GDL, dry GDE, and wet GDE

We pre-flooded the GDEs before carrying out the $CO_2$ electrolysis run (**Figure S11**) to take the differences in flooding behavior of a dry GDE and a wet GDE into account. This ensures that the effects of the cathode potential on $\Delta p^*$ the $CO_2$ electrolysis run are isolated from the effect of residual liquid saturation, $S_L^0$. The flooding behavior of porous gas diffusion media can be characterized by a capillary pressure curve (**Figure S12**). Typically, the flooding of hydrophobic pores with a non-wetting fluid requires higher capillary pressures when it is carried out the first time (Dry GDE) in comparison to subsequent flooding cycles (Wet GDE). This phenomenon can be explained through residual water being present in the network, which reduces the mechanical work necessary to force water into the hydrophobic pores during subsequent cycles.[6, 7]

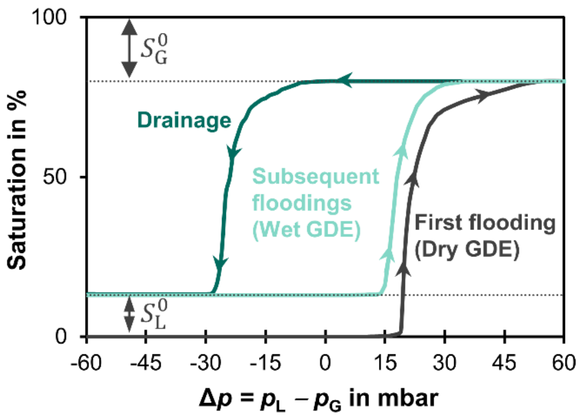


**Figure S12**: Qualitative capillary pressure curves for first flooding (Dry GDE) and subsequent flooding cycles (Wet GDE). The liquid saturation increases with increasing liquid pressure until it reaches the residual gas saturation, $S_G^0$. Reducing the liquid pressure leads to a drainage of the pore volume and a reduction of the liquid saturation down to the residual liquid saturation, $S_L^0$.[7] Adapted from Figure 11 of Gostik *et al.*.[6]

The flow-by pressure window $\Delta p^*$ of the wet GDE constitutes a worst case assumption for the flooding resistance of a GDL material (**Figure S13**). In comparison with the uncoated GDL, the deposition of the CL reduces the liquid breakthrough pressure $\Delta p_L^*$ because the CL consists of the more hydrophilic materials silver and Nafion.[1] The pre-flooding shifts $\Delta p_L^*$ of the initially dry GDEs to even more negative values and leads to a narrower $\Delta p^*$ for most samples (wet GDE).

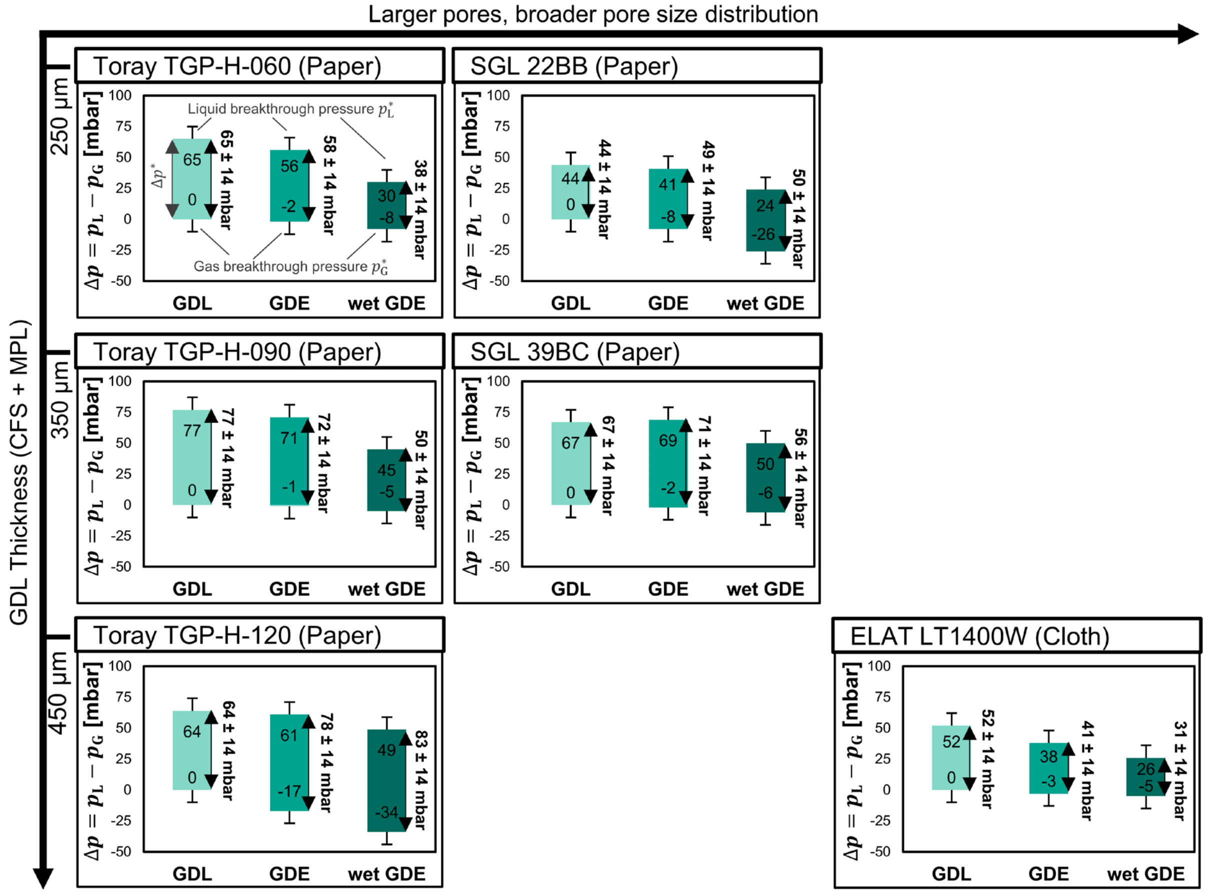


**Figure S13**: Change of the flooding resistance due to application of CL and pre-flooding of the GDE. Determination of flow-by pressure window (at open circuit), $\Delta p^* = \Delta p_L^* - \Delta p_G^*$, for uncoated GDL (MPL + CFS), coated GDE (CL + MPL + CFS), and pre-flooded/wet GDE. Upper limit of bar chart: Liquid breakthrough pressure, $\Delta p_L^*$. Lower limit: Gas breakthrough pressure, $\Delta p_G^*$. The gas breakthrough pressure limit of the uncoated GDL samples was not measured; we assume that it was 0 mbar. The arrows next to the bar charts indicate the corresponding flow-by pressure window, $\Delta p^*$. The GDL data and (dry) GDE data are identical with the data of our previous publication.[1] The listed values for the wet GDE are based on measurements of a single sample. For the breakthrough pressures, we estimated errors of $\sigma_{p_G^*} = \pm 10$ mbar and $\sigma_{p_L^*} = \pm 10$ mbar of all GDEs based on the work of Mortazavi *et al.*[8] The error of the flow-by pressure window, $\Delta p^*$, was estimated with the Gaussian error propagation: $\sigma_{\Delta p^*} = \left(\sigma_{\Delta p_L^*}^2 + \sigma_{\Delta p_G^*}^2\right)^{0.5}$.

## 7.3 Liquid breakthrough as a function of differential pressure and current density

We calculated the liquid breakthrough flow rate, $F_{\mathrm{L}}$, with a mass balance around the gas compartment and with the data of the gas–liquid phase sensor attached to the outlet of the gas compartment (**Figure S14**).

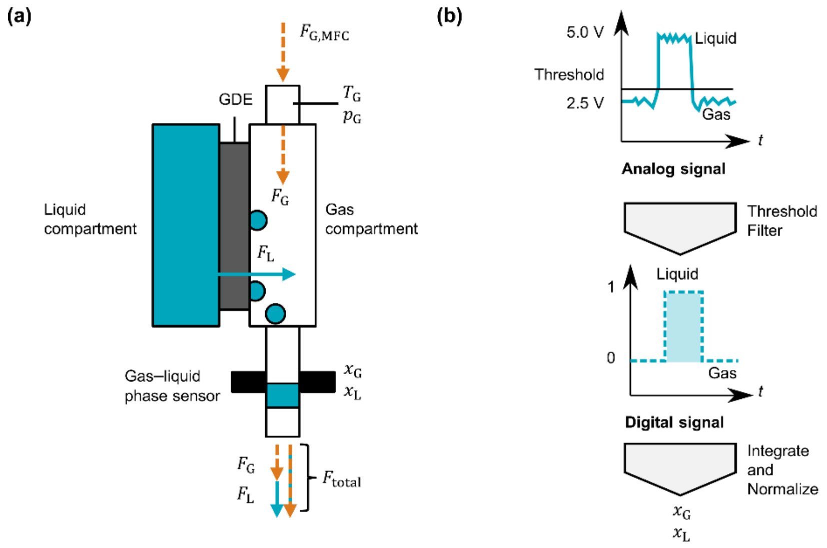


**Figure S14**: **(a)** Determination of liquid breakthrough flow rate, $F_{\mathrm{L}}$, with a mass balance around the gas compartment. The gas feed flow rate at normal conditions is $F_{\mathrm{G,MFC}}$. The feed gas has the pressure $p_{\mathrm{G}}$, and the temperature $T_{\mathrm{G}}$, and the volumetric flow rate $F_{\mathrm{G}}$ at the reactor inlet. The total two-phase flow rate is $F_{\mathrm{total}}$. The volumetric gas and liquid fractions are $x_{\mathrm{G}}$ and $x_{\mathrm{L}}$. **(b)** Measurement of $x_{\mathrm{G}}$ and $x_{\mathrm{L}}$ with the gas–liquid sensor at the gas compartment outlet.

Electrolyte that broke through the GDE left through the tubing at the bottom of the reactor (**Figure S14 a**). The two-phase flow in the outlet tube consists of liquid electrolyte and the product gas of the reactor. The volumetric gas fraction, $x_{\mathrm{G}}$, and the volumetric liquid fraction, $x_{\mathrm{L}}$, add up to 100% (S6). The volumetric gas flow rate, $F_{\mathrm{G}}$, and the volumetric liquid flow rate, $F_{\mathrm{L}}$, add up to the total volumetric flow rate, $F_{\mathrm{total}}$, in mL min$^{-1}$ (S7). The value of $F_{\mathrm{G}}$ and $F_{\mathrm{total}}$ are connected through $x_{\mathrm{G}}$ (S8).

$$1 = x_{\mathrm{G}} + x_{\mathrm{L}} \tag{S6}$$

$$F_{\mathrm{total}} = F_{\mathrm{G}} + F_{\mathrm{L}} \tag{S7}$$

$$F_{\mathrm{G}} = F_{\mathrm{total}} \cdot x_{\mathrm{G}} \tag{S8}$$

We can solve the equation system for the desired $F_{\mathrm{L}}$ by bringing $F_{\mathrm{L}}$ to the left side of (S7). We then substitute $F_{\mathrm{total}}$ with (S8) and exclude $F_{\mathrm{G}}$ to receive (S9). The values of $F_{\mathrm{G}}$ and $x_{\mathrm{G}}$ are available through experimental data.

$$F_{\mathrm{L}} = F_{\mathrm{total}} - F_{\mathrm{G}} = \frac{F_{\mathrm{G}}}{x_{\mathrm{G}}} - F_{\mathrm{G}} = F_{\mathrm{G}} \cdot \left(\frac{1}{x_{\mathrm{G}}} - 1\right) \tag{S9}$$

The gas flow rate, $F_{\mathrm{G}}$, in the outlet tube is determined from the gas feed flow rate, $F_{\mathrm{G,MFC}}$. We can assume the $F_{\mathrm{G,MFC}}$ changes little while passing through the reactor because $CO_2$ is supplied in large excess. The $CO_2$R reaction would have little effect on the volumetric flow rate because each converted molecule of $CO_2$ is replaced with a molecule of CO. We assume that the HER does not contribute to $F_{\mathrm{G}}$ because we observed the formation of hydrogen bubbles at the liquid side of the CL. Any evolved hydrogen, therefore, should leave the reactor through the liquid outlet instead of the gas outlet. The feed flow rate, $F_{\mathrm{G,MFC}}$, has a value of 50 mLn min$^{-1}$ (normal conditions: 0 °C,

1.013 bar (a)). We adjusted this flow rate to the conditions in the reactor with the ideal gas equation. We assumed that the gas temperature, $T_{\mathrm{G}}$, remained constant at the feed temperature of 20 °C. The gas pressure, $p_{\mathrm{G}}$, was measured with the gas sensor in front of the reactor. The data are available in **accompanying Excel sheet**.

The volumetric gas fraction, $x_{\mathrm{G}}$, in the outlet tube can be estimated from the phase sensor data (**Figure S14 b**). A liquid slug that passes through the sensor, causes a peak in the analog output voltage. We converted the analog data signals to digital data signals by applying a threshold filter. A value of 0 in for the digital data indicates that gas is present at the sensor; a value of 1 indicates that liquid is present. We integrated the sensor data for each experimental setting (current density step and differential pressure) to determine the averaged value of $x_{\mathrm{G}}$.

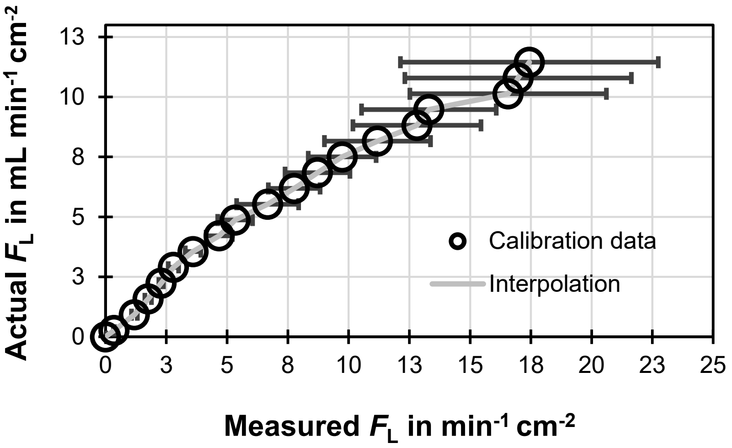


**Figure S15**: Gas–liquid sensor calibration of the liquid breakthrough flow rate, $F_{\mathrm{L}}$. The measured $F_{\mathrm{L}}$ was determined with the sensors and calibrated against the actual liquid flow rate. The markers represent the average measured $F_{\mathrm{L}}$, the error bars represent the standard error determined through three independent calibration runs. The data acquisition rate of the sensor was 20 Hz. We used a linear interpolation between the data points to correct all data from the $CO_2$ electrolysis experiments.

The measured $F_{\mathrm{L}}$ determined with (S9) was corrected with a non-linear calibration curve (**Figure S15**). This was necessary because the actual and the measured $F_{\mathrm{L}}$ start deviating from each other at higher flow rates.
This deviation can be attributed to a number of phenomena:

- The data acquisition rate of 20 Hz limits the signal's time resolution
- The liquid slugs deviate from an ideal cylinder shape. This makes the volume calculation inaccurate.
- The two-phase flow is less steady at high flow rates.

For these reasons, the error starts increasing at higher flow rates, which makes it more difficult to distinguish higher flow rates from each other. At low flow rates, for example, we calculate a measured $F_{\mathrm{L}}$ of 2.9 mL min$^{-1}$ cm$^{-2}$ from (S9), which corresponds to an actual flow rate of 2.8 ± 0.2 mL min$^{-1}$ cm$^{-2}$. If we compare this to a higher flow rate of 15.9 mL min$^{-1}$ cm$^{-2}$, this then corresponds to 10.0 ± 3.9 mL min$^{-1}$ cm$^{-2}$.

Our values for $F_{\mathrm{L}}$ are a bit larger, but in a similar order of magnitude as in the study on the flooding behavior of SGL 39BC reported by Mot *et al.* (**Table S4**).[9] A possible explanation for the different values for $F_{\mathrm{L}}$ might be the different techniques used to measure $\Delta p$. De Mot *et al.* determined $\Delta p$ by comparing the pressure of the gas and the liquid inlet line. This measurement technique exaggerates the $\Delta p$ between the gas and the liquid compartment because it neglects the hydrodynamic pressure drops in the gas and in the liquid feed lines. The reported $F_{\mathrm{L}}$, therefore, might correspond to a lower value of $\Delta p$ than 30 mbar.

**Table S4**: Comparison of liquid breakthrough flow rates with literature.

| Data set | De Mot *et al.*[9] | This work |
|---|---|---|
| ***Electrode parameters*** | | |
| CL composition | 70 wt% Sn, 30 wt% Nafion | 80 wt% Ag, 20 wt% Nafion |
| CL loading | 0.75 mg Sn $cm^{-2}$ | 1 mg Ag $cm^{-2}$ |
| GDL substrate | SGL 39BC | SGL 39BC |
| Electrode area | 16 $cm^2$ | 3.8 $cm^2$ |
| ***Process parameters*** | | |
| Current density | 100 mA $cm^{-2}$ | 100 mA $cm^{-2}$ |
| $\Delta p$ | 30 mbar | 30 mbar |
| $\Delta p$ measurement technique | inline, between inlets | directly between cell compartments |
| ***Resulting liquid breakthrough flow rate*** | | |
| $F_L$ | 0.1 mL $min^{-1}$ $cm^{-2}$ | 0.9 ± 0.12 mL $min^{-1}$ $cm^{-2}$ |
| $F_L$ measurement technique | weighing | inline gas–liquid phase sensor |

The resulting $F_L$ is shown in **Figure S16** for the different materials in dependence of differential pressure, $\Delta p$, and cathode potential.

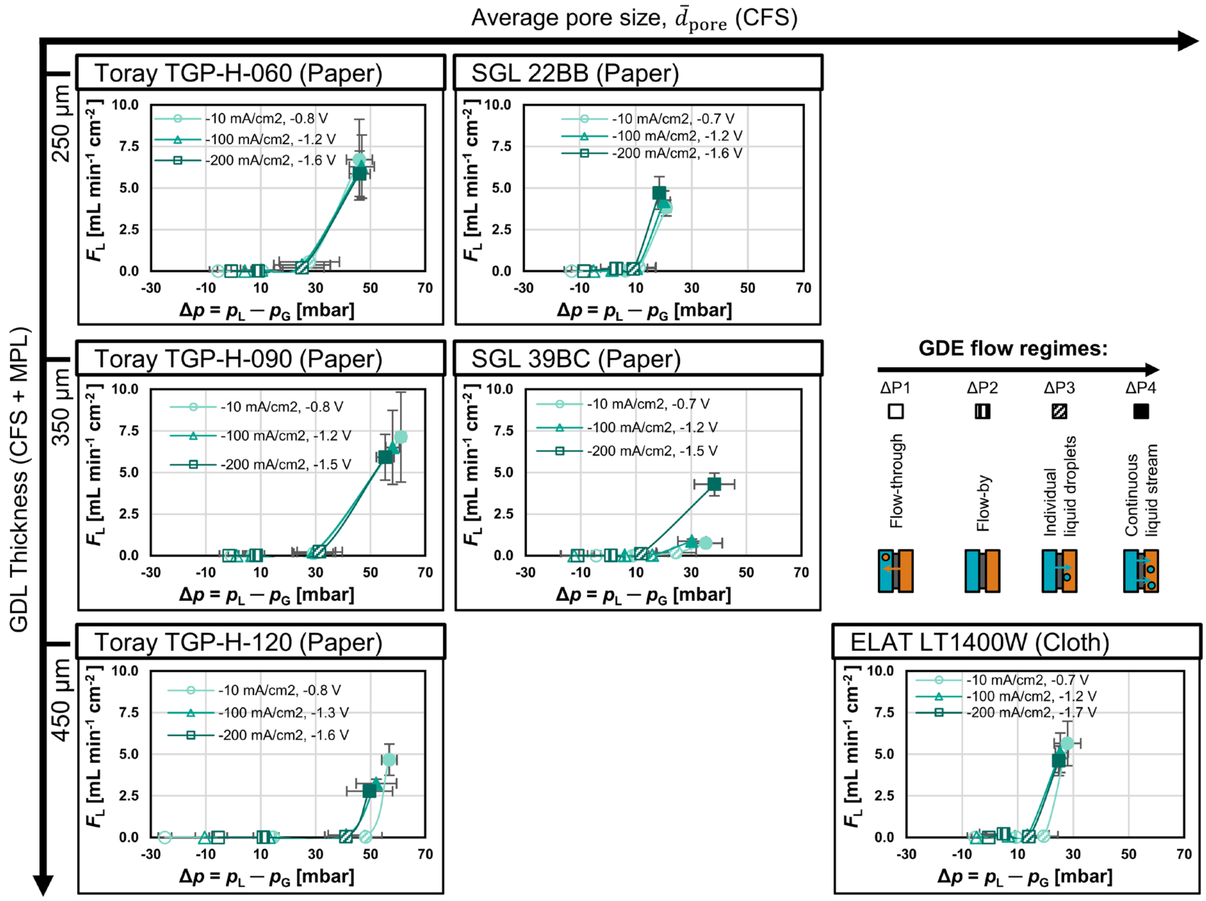


**Figure S16**: Liquid breakthrough flow rates $F_L$ as a function of differential pressure $\Delta p$. The series of markers corresponds to the current densities −10, −100, and −200 mA $cm^{-2}$. The *y*-axis error bars represent the estimated error of the calibration curve (**Figure S15**). The *x*-axis error bars represent the sample standard deviation of the recorded differential pressure. The transition from individual liquid droplets to a continuous liquid breaking through occurred around 1 mL $min^{-1}$ $cm^{-2}$.

## 7.4 Cathode potential as a function of differential pressure and current density

The cathode potentials, $E_{\mathrm{Cathode}}$, recorded during the $CO_2$ electrolysis experiments with varying current density and differential pressure are shown in **Figure S17**.

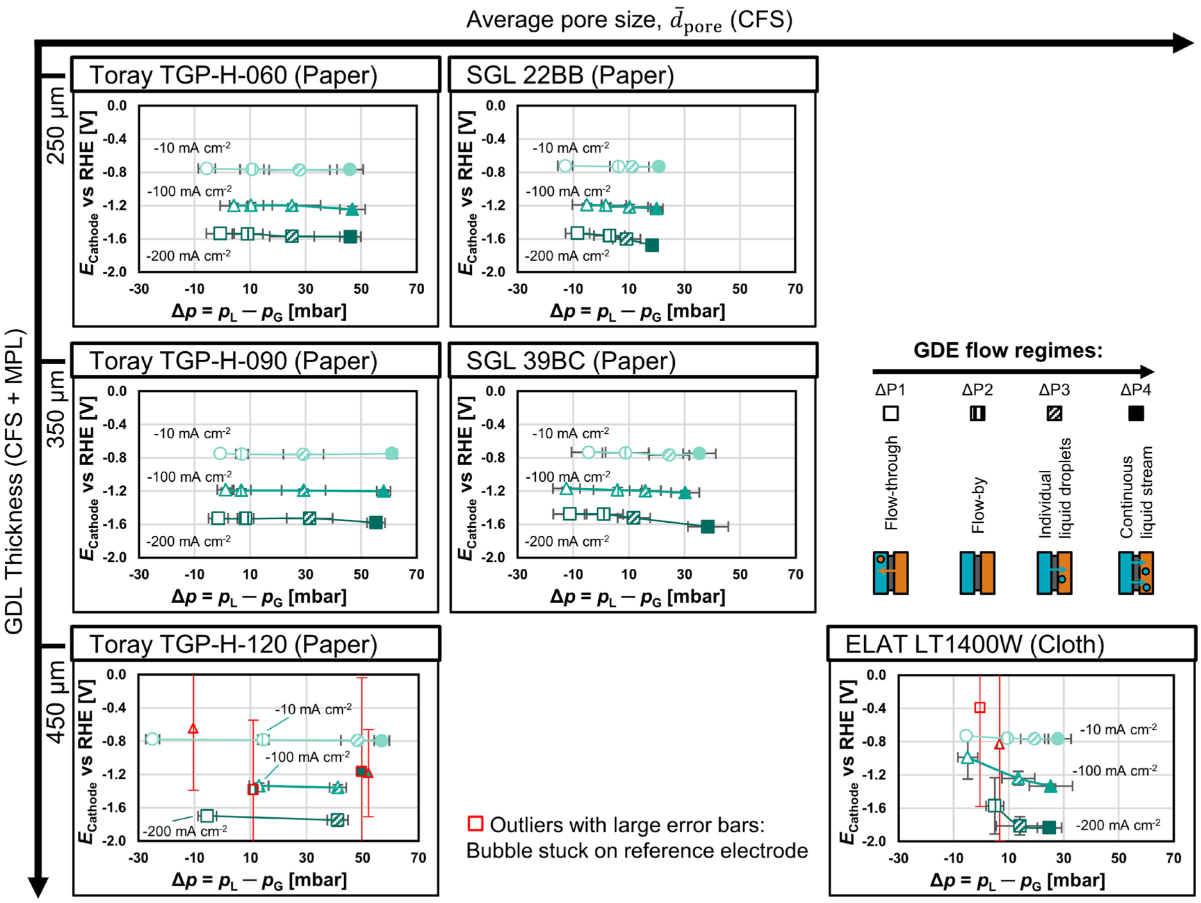


**Figure S17**: Cathode potential, $E_{\mathrm{Cathode}}$, as a function of differential pressure, $\Delta p$. The data series correspond to the current densities −10 (O), −100 (Δ), and −200 mA cm$^{-2}$ (□) from lighter to darker color. The marker filling indicates the GDE flow regime. The *y*-axis error bars represent the sample standard deviation of the recorded potential. Some measurements of TGP-H-120 and LT1400W suffered from high fluctuations in the cathode potential due to gas bubbles blocking the reference electrode. The marker outline of these outliers is marked in red. The *x*-axis error bars represent the sample standard deviation of the recorded $\Delta p$.

With increasing current density, $E_{\mathrm{Cathode}}$ increases due to the electrochemical overpotential of the $CO_2$R and the HER taking place. For most materials, $E_{\mathrm{Cathode}}$, does not depend strongly on $\Delta p$. For the ELAT LT1400W carbon cloth, however, the potential becomes more negative with increasing $\Delta p$. At a current density of −200 mA cm$^{-2}$, for example, $E_{\mathrm{Cathode}}$ drops from –1.57 V vs RHE at +5 mbar to –1.83 V vs RHE at +25 mbar. We think this phenomenon can be explained through by the flexibility of the cloth, which makes it deform at higher liquid overpressures. This mechanical deformation then increases the distance between the reference electrode and the cathode, which leads to an uncompensated ohmic resistance. The other materials are less flexible because the binder in the carbon paper makes them rigid. We discuss this effect in more detail in the next section (**Figure S20**).

## 7.5 Stability during $CO_2$ electrolysis with varying differential pressures

The electrochemical performance of GDEs can decrease over time due to mechanisms like catalyst leaching or deactivation, salt formation, and/or loss of GDE hydrophobicity. We tested the stability of our GDE materials to ensure their performance was sufficiently stable during the 4.5 h of our main $CO_2$ electrolysis run. In a previous publication, we showed that the GDE based on SGL 39BC did not show any significant loss in the Faradaic efficiency for CO, $FE_{CO}$, over a run time of 2 h at a current density of 190 mA cm$^{-2}$.[1] In this publication, we present additional results for GDEs based on TGP-H-060 and TGP-H-120. We tested the stability of these GDEs by repeating the experiments with the parameter set of ΔP1 and 100 mA cm$^{-2}$ after completing the main $CO_2$ electrolysis run (**Figure S18**).

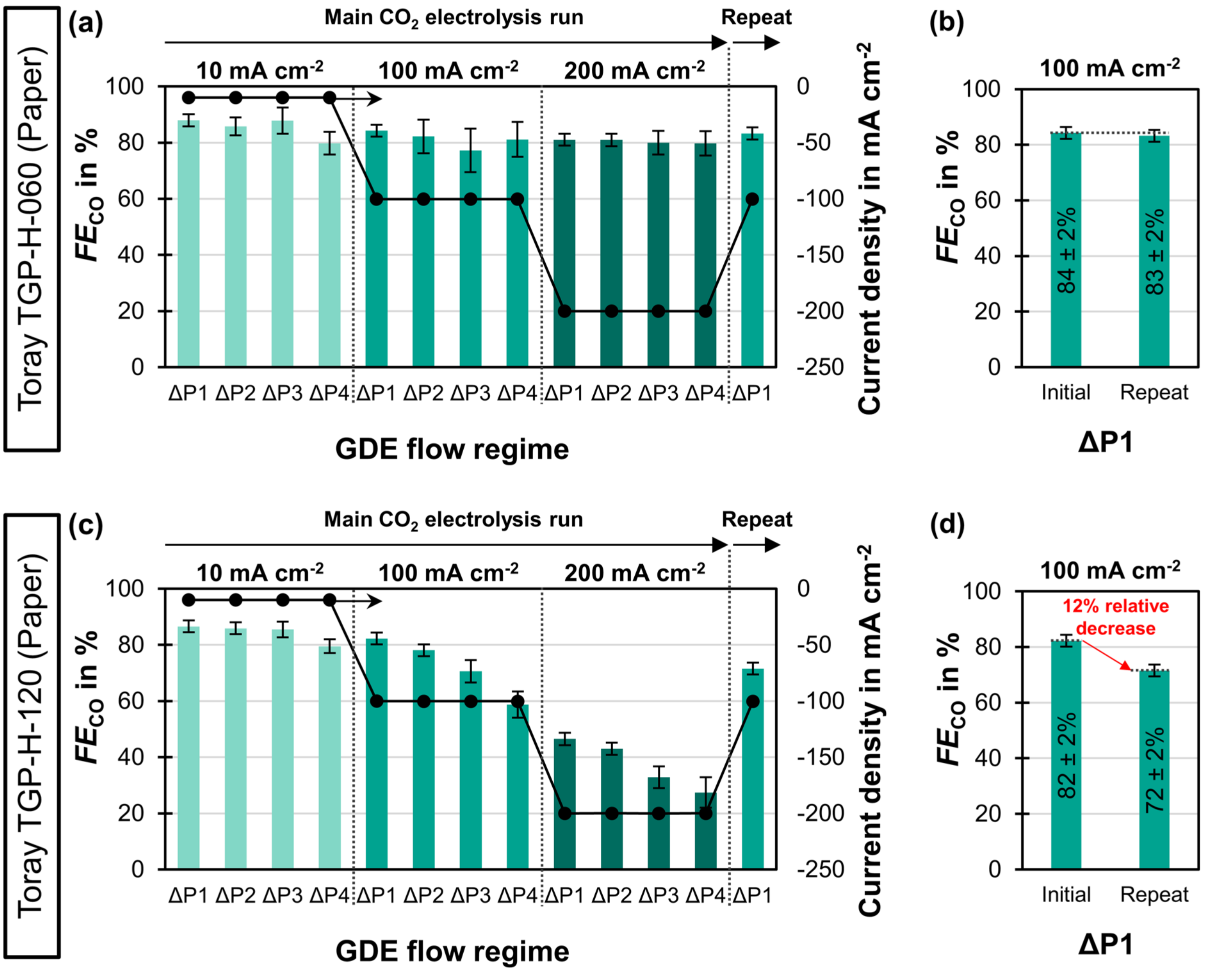


**Figure S18**: Stability test with TGP-H-060 and TGP-H-120 carbon papers. After the main $CO_2$ electrolysis run (duration of 4.5 h), we repeated the parameter set of ΔP1 and 100 mA cm$^{-2}$ (duration of 7 min). **TGP-H-060: (a)** Faradaic efficiency, $FE_{CO}$, for all parameter sets (combination of current density and GDE flow regime ΔP) of the main $CO_2$ electrolysis run and the repeated experiment. **(b)** Comparison of $FE_{CO}$ at ΔP1 and 100 mA cm$^{-2}$ between main run (initial) and repeat experiment. **TGP-H-120: (c)** $FE_{CO}$ for all parameter sets of the main $CO_2$ electrolysis run and the repeat experiment. **(d)** Comparison between main run (initial) and repeat experiment.

For TGP-H-060, the comparison of the initial $FE_{CO}$ recorded at ΔP1 and 100 mA cm$^{-2}$ shows that there was no significant reduction in $CO_2$ reduction performance (**Figure S18 b**). In contrast, TGP-H-120 showed a slight performance loss during the 4.5 h electrolysis run. This can be seen in the drop of $FE_{CO}$ from 82% for the initial experiment to 72% for the repeat experiment (**Figure S18 d**), which corresponds to a relative decrease of 12%.

These results implies that the effect of the GDE flow regime is partially convoluted with this loss in performance, especially at the highest current density.
A possible explanation for the deactivation of the GDE could be the leaching of Ag from the catalyst layer into the electrolyte. Another explanation could be the contamination of the cathode GDE with other metals, which catalyze the HER reaction, such as Fe or Ni.[5] We used ICP-OES to measure the concentrations of metal ions in the electrolyte after the electrolysis procedure to test these hypotheses (**Table S5**).

**Table S5**: Leaching and contamination study: Metal concentrations in 1 M $KHCO_3$ electrolyte determined with ICP-OES. Detection limit: 0.01 mg $L^{-1}$.

| Experiment | Run time | Ag | Fe | Ni |
|---|---|---|---|---|
| **Blank** | 0 h | < 0.01 mg $L^{-1}$ | 0.02 mg $L^{-1}$ | < 0.01 mg $L^{-1}$ |
| **TGP-H-060** | 4.5 h + 0.5 h | < 0.01 mg $L^{-1}$ | 0.08 mg $L^{-1}$ | 0.09 mg $L^{-1}$ |
| **TGP-H-120** | 4.5 h + 0.5 h | < 0.01 mg $L^{-1}$ | 0.14 mg $L^{-1}$ | 0.11 mg $L^{-1}$ |

The leaching of Ag probably does not significantly contribute to the performance decrease of the GDE. Our data shows that Ag concentration in the electrolyte remained below the detection limit (**Table S5**). This means that in the worst case, there might be up to 0.01 mg $L^{-1}$ Ag in the 60 mL of electrolyte. We can estimate that this would correspond up to 0.016% of the Ag on the GDE being leached, which has an area of 3.8 $cm^2$ and a loading of 1 mg $cm^{-2}$. The leaching of Ag seems an unlikely explanation for the deactivation, however, because we would expect the leaching rates and the resulting deactivation to be similar for the two GDE samples. This is evidently not the case because TGP-H-120 exhibited a stronger performance decrease than TGP-H-060 (**Figure S18**).

The contamination with Fe or Ni, similarly, also seems an unlikely explanation for the degradation because it can not explain the difference between the two samples. We measured a slight increase in the Fe and Ni concentrations compared to the blank sample (**Table S5**). These contaminants likely originate from the contact of the electrolyte with the tubes, fittings, and valves made from stainless steel (Grade 316). We did not detect any Fe or Ni on the surface of the TGP-H-060 sample with XPS (results not shown).

Another possible explanation for the loss of $CO_2$ reduction performance of TGP-H-120 could be the (electro-) chemical degradation of the CFS, which is associated with HER and can lead to a loss of hydrophobicity (lower contact angle).[10] This phenomenon could have led to additional flooding of the GDE and a decrease in $CO_2$ diffusivity. However, we did not measure any significant change of the contact angle due to the $CO_2$ electrolysis run to support this hypothesis (**Table S5**).

**Table S6**: Comparison of static contact angle ± standard error before electrolysis and after $CO_2$ electrolysis run. The data was originally published in our previous publication.[1]

| Experiment | CFS before | CFS after |
|---|---|---|
| **TGP-H-060** | 147 ± 1.0 | 148 ± 0.6 |
| **TGP-H-120** | 148 ± 0.4 | 149 ± 0.4 |

The performance loss of TGP-H-120 (**Figure S18**) might be due to a change inside of the pore network. The residual liquid saturation might have increased over the course of the experiment due to salt formation or loss of hydrophobicity inside of the pores. Precipitated salt could have also blocked pores. These phenomena might have decreased the $CO_2$ diffusion for the repeat experiment. This kind of deactivation mechanism could be investigated with X-ray tomographic microscopy[11, 12] in future studies.

# 8. $CO_2$ electrolysis performance test with carbon cloth

We conducted a performance test of the carbon cloth GDE for 120 h with a $\Delta p$ of up to 120 mbar. This test should be able to estimate the local performance at the bottom of a $CO_2$ electrolyzer, which is subject to a hydrostatic pressure difference between catholyte and gas phase. At this high liquid overpressure, electrolyte breaks through the GDE continuously. We used the sample GDE sample based on the ELAT LT1400W carbon cloth that we previously tested in our $CO_2$ electrolysis run with varying $\Delta p$ and current density (**Section 7**). We used a check valve with a cracking pressure of 69 mbar at the outlet of the gas compartment. The complete data set is available in the sheet "**ELAT flow through run**" in the **accompanying Excel file**.

## 8.1 Faradaic efficiency and cathode potential as a function of differential pressure

To determine the average $FE_{\mathrm{CO}}$ over the height of a reactor with mixed flow regime (**Figure S19 a**), we varied $\Delta p$ from −6 mbar to +109 mbar while the potentiostat was set to a galvanostatic current density step of 200 mA cm$^{-2}$. The $FE_{\mathrm{CO}}$ ranged from 80% to 60% over the different flow regimes (flow-by to continuous liquid breakthrough). The data points in the flow-by regime ($\Delta p = -6$ mbar) correspond to the top of a hypothetical reactor; the data points with continuous liquid flow-through ($\Delta p =$ 109 mbar) correspond to the bottom of a hypothetical reactor. We determined the weighted average $FE_{\mathrm{CO}}$ of 69% by integrating the $FE_{\mathrm{CO}}$ over the corresponding range of $\Delta p$. The calculations are included in the sheet "**Average FE_CO cloth**" of the Excel file.

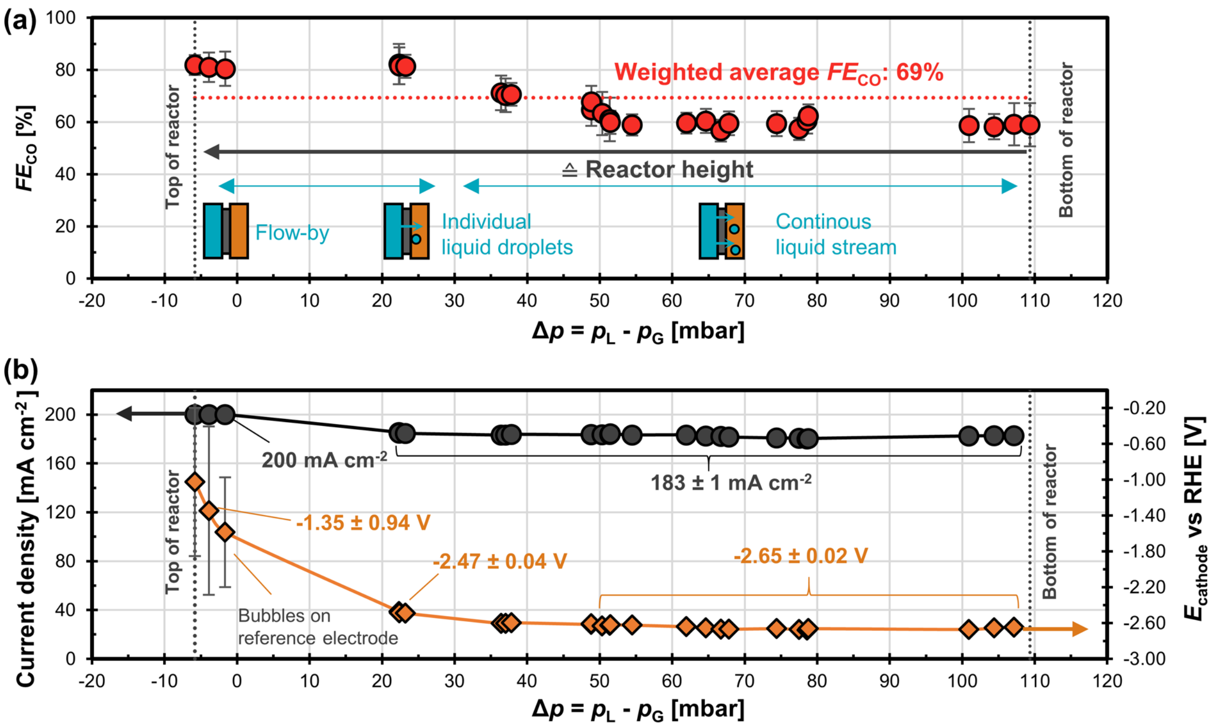


**Figure S19**: **(a)** Larger version of **Figure 7 a**: Faradaic efficiency for CO, $FE_{\mathrm{CO}}$, as a function of differential pressure, $\Delta p$. The cell potential was constant at 10 V (Potentiostat limit). The average $FE_{\mathrm{CO}}$ was determined by integrating $FE_{\mathrm{CO}}$ numerically over the $\Delta p$ range. The blue arrows below the $FE_{\mathrm{CO}}$ data points indicate the GDE flow regime. **(b)** Current density (left axis) and cathode potential, $E_{\mathrm{cathode}}$, as a function of $\Delta p$. The large error bars of the first three data points were caused by bubble formation between the cathode surface and the reference electrode.

While we were able to reach the current density set point of 200 mA cm$^{-2}$ in the flow-by regime, the potentiostat only achieved 183 mA cm$^{-2}$ at higher $\Delta p$ (**Figure S19 b**). The current density was limited because cathode potential

increased significantly beyond $\Delta p \geq 22$ mbar, which let the potentiostat reach the cell potential limit of 10 V. It is important to understand the source of this increased cathode overpotential to improve the energy efficiency of the process.

The decreasing $E_{\mathrm{cathode}}$ (**Figure S19 b**) might impact $FE_{\mathrm{CO}}$ in addition to the effects of $\Delta p$. According to a kinetic study of an Ag electrocatalyst in $KHCO_3$ by Jaramillo *et al.*,[13] higher cathode overpotentials can favor the kinetics of HER. Therefore, it is possible that the decreasing $E_{\mathrm{cathode}}$ changes the relative kinetics of the $CO_2$R and HER. These kinetic effects are convoluted with mass transfer limitation effects at high current density making it difficult to assess their relative importance. The data in **Figure S19** suggest that there is no significant correlation between $E_{\mathrm{cathode}}$ and $FE_{\mathrm{CO}}$ for the investigated GDE. For example, $FE_{\mathrm{CO}}$ remains constant while $E_{\mathrm{cathode}}$ drops from –1.35 V to –2.47 V ($\Delta p = -5$ mbar vs. $\Delta p = +23$ mbar). Further, the drop in $FE_{\mathrm{CO}}$ from 71% to 61% occurs without a change in $E_{\mathrm{cathode}}$ ($\Delta p = +36$ mbar vs. $\Delta p = +51$ mbar). Therefore, we can assume that $FE_{\mathrm{CO}}$ depends predominantly on the $CO_2$ mass transfer, which is a function of $\Delta p$ and the saturation behavior of the GDE's pore network.

The decrease in cathode potential is caused by the deformation of the flexible carbon cloth through liquid overpressure. The cathode potential was measured with an Ag/AgCl micro-reference electrode, which was placed with a gap of 0.5 mm from the GDE surface (**Figure S20 a**). The cathode potential was compensated for the ohmic resistance arising in this small gap. We observed that the carbon cloth bulged at higher $\Delta p$ due to the mechanical pressure from the liquid side (**Figure S20 b**). The bulging increased the gap between the cathode and the reference electrode, which introduced an uncompensated potential drop (**Figure S20 c**). The effect of this increased gap on the apparent cathode potential is substantial because of the low electrolyte conductivity of $\kappa = 0.077$ S cm$^{-1}$ (**Figure S20 d**).[14] For example, the gap of 4 mm would increase the apparent cathode potential by 0.95 V at 183 mA cm$^{-2}$. Therefore, the mechanical deformation can explain the more negative cathode potential observed at higher values for $\Delta p$ in **Figure S17** and **Figure S19**. While the bulging of the cloth GDE introduces a systematic error into measured cathode potential of our lab experiments, this phenomenon does not inherently limit the energy efficiency of this material for application. The flexible cloth can be supported mechanically with a rigid mesh and/or a gas flow field, which also serves as a current collector.

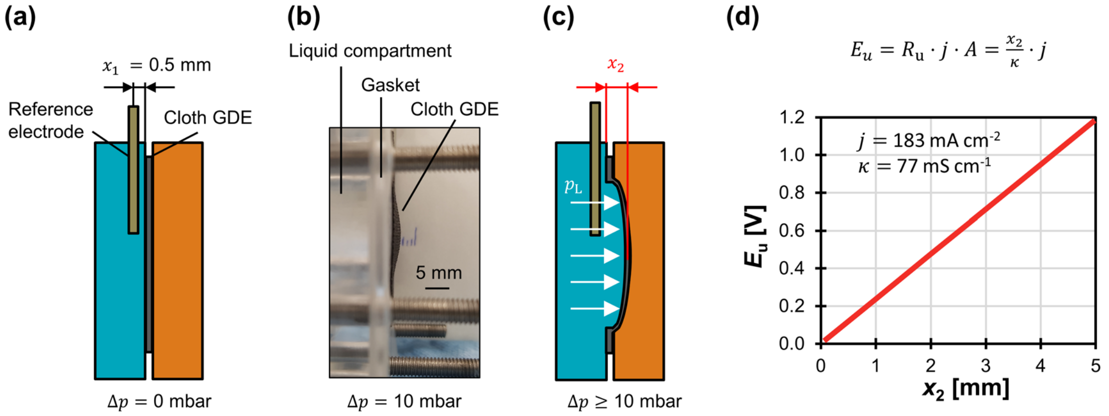


**Figure S20**: Cloth deformation increases apparent cathode overpotential. **(a)** At $\Delta p = 0$ mbar, the ohmic resistance arising in the 0.5 mm gap between cathode GDE and the micro-reference electrode is compensated. **(b)** Disassembled cell after imposing a liquid overpressure of $\Delta p = 10$ mbar: The cloth bulged by about 2 mm. **(c)** Liquid overpressures introduce an unknown additional gap, $x_2$, between the reference electrode and the cathode. **(d)** Uncompensated potential, $E_{\mathrm{u}}$, as a function of the additional gap width, $x_2$. The 1 M $KHCO_3$ had ionic conductivity, conductivity of $\kappa = 0.077$ S cm$^{-1}$.[14]

## 8.2 Performance test with continuous liquid breakthrough

To carry out the more stringent $CO_2$ performance test (**Figure 7 b**), the potentiostat was set to a galvanostatic current density step of 200 mA cm$^{-2}$ continuously for 125 h. We were, however, only able to reach a current density of 180 – 193 mA cm$^{-2}$ because of the cell potential limit of 10 V (**Figure S21**). The $\Delta p$ ranged from 80 to 100 mbar, which is discussed further below (**Figure S23**).

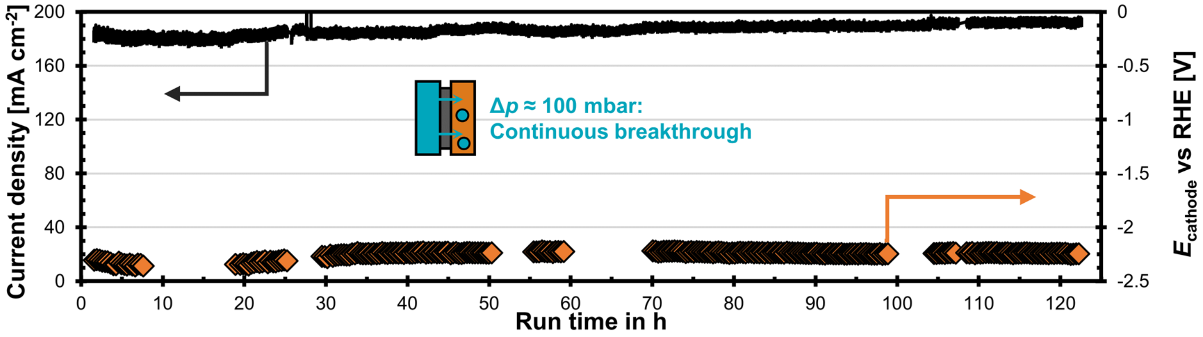


**Figure S21**: $CO_2$R performance test of ELAT LT1400W carbon cloth GDE exhibiting continuous catholyte breakthrough: $\Delta p$ ranged from 80 to 120 mbar: Current density (left axis) and cathode potential, $E_{\mathrm{cathode}}$, over the run time.

The $FE_{\mathrm{CO}}$ remained stable at 60 ± 4.5% for 10 h (**Figure S22 a**). We had to depressurize and restart the electrolysis system three times because of technical issues (Labview program crashed, had to switch to new $CO_2$ tank). These restarts had a negative effect on $FE_{\mathrm{CO}}$. It is unclear to us why it took the $FE_{\mathrm{CO}}$ several hours to re-stabilize to a steady state value because it only took several minutes to re-pressurize the electrolyzer after a restart. We highlighted the data points for which the system was still re-stabilizing.

The system was operating at an average $FE_{\mathrm{CO}}$ of 55 ± 6.8% at the end of the performance test (**Figure S22 a**). We hypothesize that this reduction in performance was not due to a change in the GDE, but due to the reduction of the purge flow rate during the run (**Figure S22 c**), which lead to an increased crossover of gaseous products within the system. The errors in Faradaic efficiency increase after 20 h run time because the reduction of the purge flow rate lead to a stronger fluctuation of the product gas flow rate measured at the mass flow meter (**Figure S6**).

We can support this crossover hypothesis by comparing the Faradaic efficiency for the cathode reactions ($CO_2$R and $H_2$; **Figure S22 b**) with the anode side reactions (OER; **Figure S22 c**). For the first 20 h of the electrolysis run, the $CO_2$ purge flow rate was 80 mLn min$^{-1}$ (**Figure S22 c**). During this time the combined Faradaic efficiency on the cathode side, $FE_{\mathrm{CO+H_2}}$, add up to an average of 83 ± 6.5% (**Figure S22 b**). The Faradic efficiency on the anode side, $FE_{\mathrm{O_2}}$, has an average value of 95% ± 4.6%. The $FE$ probably does not reach 100% because a part of the product gases ($O_2$, CO, $H_2$) remains dissolved in the electrolyte and is then feed to the “wrong” electrode by the pump. This way, for example, a part of the $O_2$ produced at the anode could remain dissolved in the electrolyte and be feed to the cathode, where the oxygen reduction reaction (ORR) can take place ($O_2 + 4\ e^- \rightarrow 4\ OH^-$).[15] Because this reaction would consume part of the current at the cathode, the $FE_{\mathrm{CO+H_2}}$ would be lower than 100%.

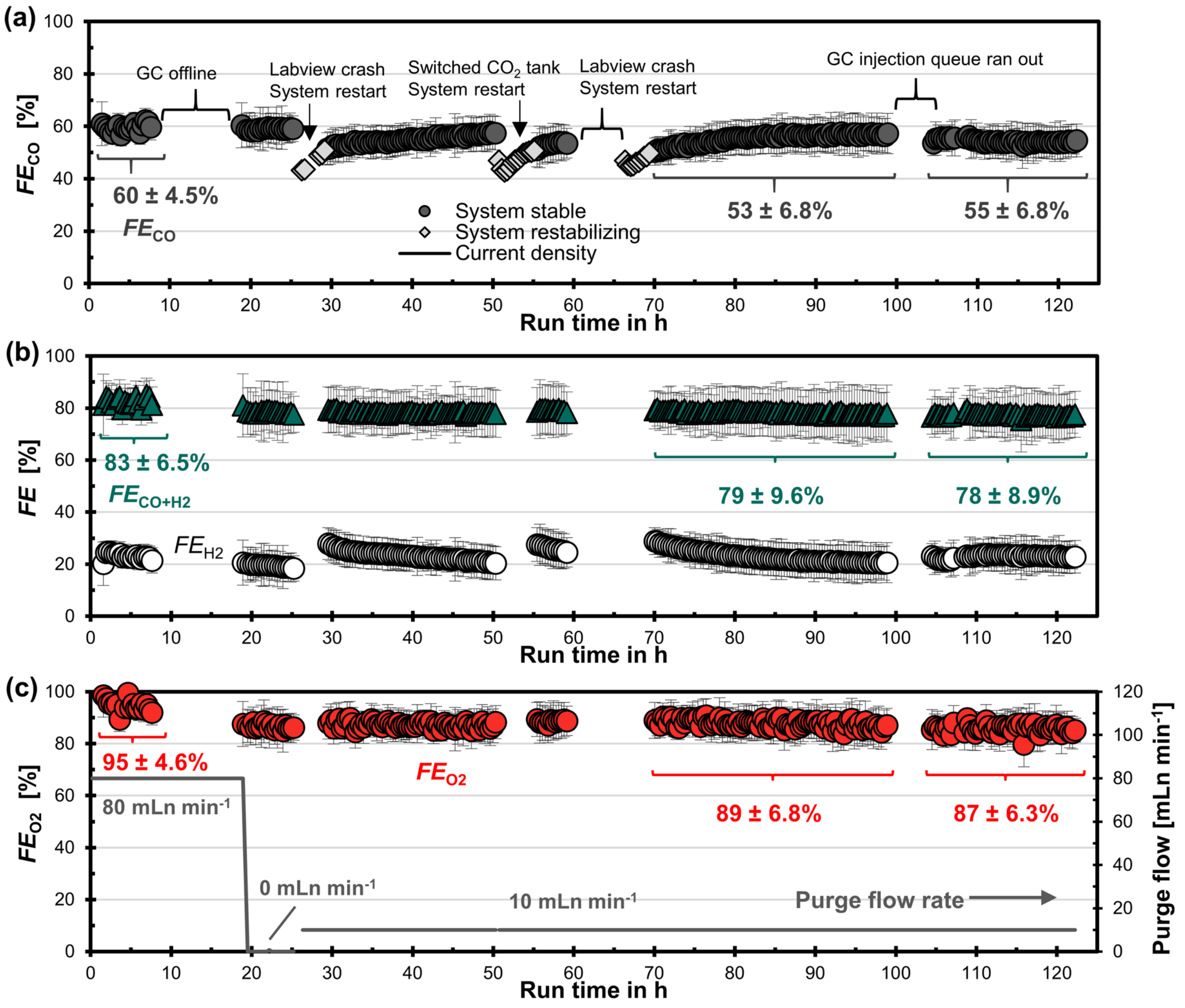


**Figure S22**: **(a)** Faradaic efficiency for CO, $FE_{\mathrm{CO}}$, (left y-axis) and current density (right y-axis) over the run time. No GC data is available for the periods when the GC was idle or when we had to restart the system. The electrolysis system required several hours to re-stabilize the $FE_{\mathrm{CO}}$ values after each restart. These non-representative data points are highlighted with grey diamond markers (◊). **(b)** Combined Faradaic efficiency for CO and $H_2$, $FE_{\mathrm{CO+H_2}}$, and $FE_{\mathrm{H_2}}$, over the run time. **(c)** Faradic efficiency for $O_2$, $FE_{\mathrm{O_2}}$, and $CO_2$ purge flow rate over run time.

After the 25 h we reduced the $CO_2$ purge flow rate to 10 mLn min$^{-1}$ to reduce the $CO_2$ consumption from the supply tank (**Figure S22 c**). This reduction purge flow increased the crossover of gaseous products in the system because dissolved product gases were being removed from the electrolyte bottle less effectively. As a consequence, the deficits in $FE$ increased for both electrodes. On the cathode side, $FE_{\mathrm{CO+H_2}}$ dropped from an average of 83 ± 6.5% to 78 ± 8.9% (**Figure S22 b**). On the anode side, $FE_{\mathrm{O_2}}$ dropped from 95 ± 6.5% to 87 ± 6.3% (**Figure S22 b**). We argue, therefore, this product gas crossover is a primary reason for the reduction in $FE_{\mathrm{CO}}$ from 60% to 55% over the course of the experiment (**Figure S22 a**). No other gaseous products were detected with the gas chromatography system ($CH_4$, $C_2H_4$, $C_2H_6$).

We analyzed a sample of the electrolyte after the run with HPLC to determine any liquid products. The average Faradaic efficiency for formic acid was 0.3%. We detected trace amounts of acetic acid. No ethanol was detected. It is not clear to us why the $FE$ deficit is not the same for both electrodes. The electrolyte pH after the experiment was 7.55, which is slightly higher than the initial pH of 7.41 (20 °C).

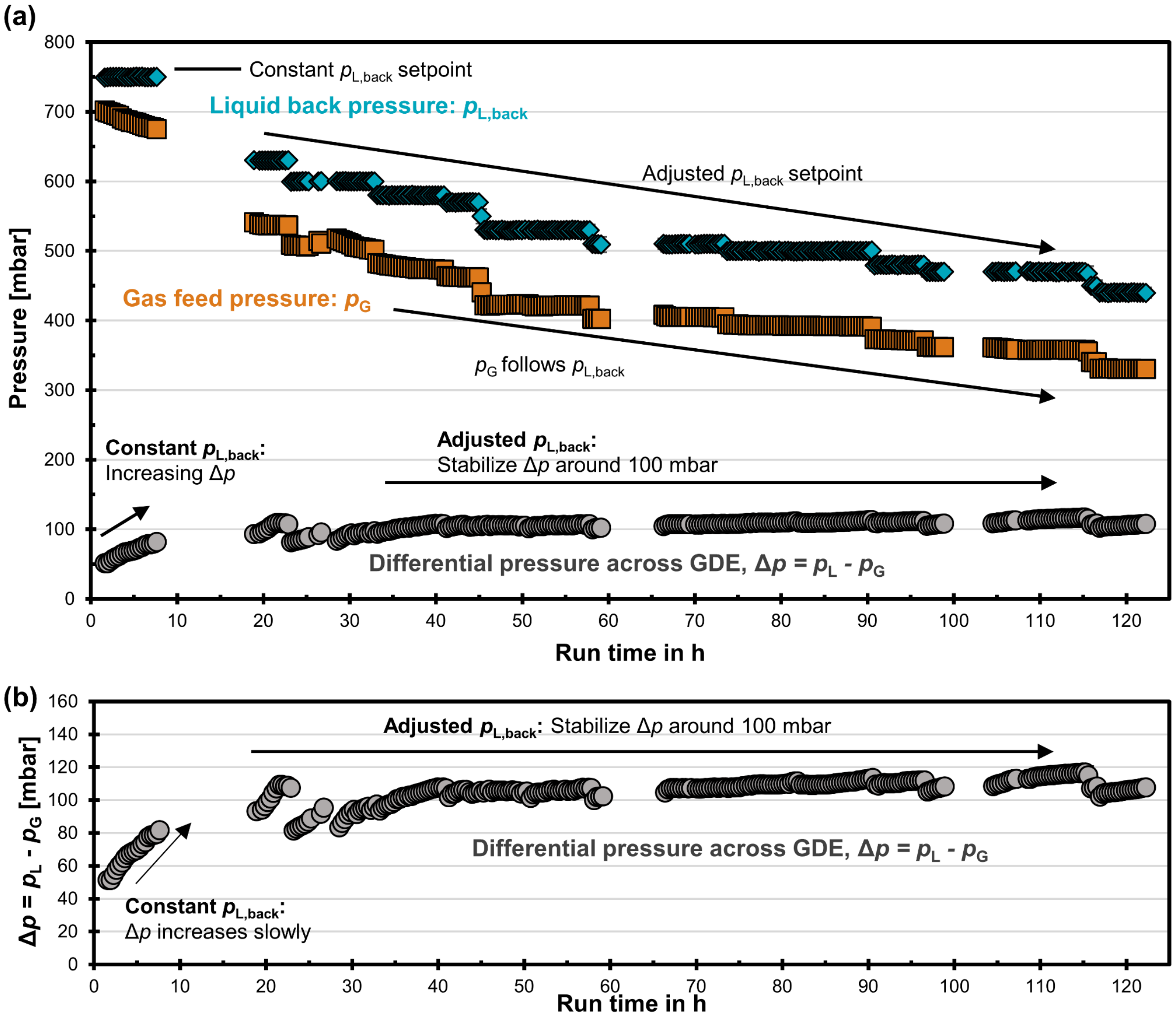


**Figure S23**: **(a)** Liquid backpressure, $p_{\mathrm{L,back}}$, at the control valve, gas feed pressure, $p_{\mathrm{G}}$, and differential pressure across GDE, $\Delta p$, over the run time. **(b)** Detail view of differential pressure across GDE, $\Delta p$, over the run time.

For the first 10h of the experiment, we kept the setpoint of the liquid backpressure, $p_{\mathrm{L,back}}$, constant at 750 mbar (**Figure S23 a**). The gas feed pressure, $p_{\mathrm{G}}$, fell steadily during this time. This resulted in a drift in the differential pressure across the GDE, $\Delta p$, from 50 mbar to 80 mbar. We adjusted $p_{\mathrm{L,back}}$ for the last 100 h of the experiment to stabilize the drifting $\Delta p$ at 100 mbar (**Figure S23 b**). The falling $p_{\mathrm{G}}$ seems to have little effect on the $FE_{\mathrm{CO}}$ (**Figure S22 a**).

The drifting $\Delta p$ (**Figure S23**) could be explained by a reduction in the liquid permeability over the course of the experiment. This might be due to carbonate salt formation or mechanical deformation of the GDE, which blocks some pores. A reduction in liquid permeability would have reduced the liquid breakthrough flow rate at a constant $p_{\mathrm{L,back}}$, which then would have also reduced $p_{\mathrm{G}}$. We can expect the $p_{\mathrm{G}}$ to fall in this situation because a lower combined liquid and gas flow rate (**Figure S14**) has to be forced through the check valve at the outlet of the gas compartment, which results in a lower pressure drop. Unfortunately, the exact liquid breakthrough flow rate is unknown because it exceeded the range of our gas–liquid phase sensor (**Figure S15**).

## 8.3 Post-electrolysis characterization

After electrolysis, we rinsed the GDE with IPA and dried it to perform additional characterization. The SEI and BEC images (x100) show the presence of large salt crystals, which have a diameter of 10 – 50 µm (**Table S7**). They appear in medium grey scale in the BEC images because they have a higher density than the carbon background and a lower density than the Ag particles of the CL (small white dots). These crystals are carbonate salts which precipitate due to the alkaline environment at the cathode.[16, 17] They are even more clearly visible at higher magnifications (**Table S8**).

**Table S7**: Salt formation on ELAT LT1400W GDE: Scanning electron microscope images of the catalyst layer after the 125 h performance test at different magnifications (x100 – x500). **Left**: Secondary electron imaging (SEI) for morphology, **Right**: Back scattered electron composition (BEC) detector for elemental contrast imaging. Dark grey domains in the BEC images indicate the carbon-rich MPL and carbon fibers. Medium grey domains indicate carbonate salt crystals. Light grey domains indicate dispersed Ag nanoparticles in a Nafion matrix.

<table>
<tr><th>SEI (Morphology)</th><th>BEC (Composition)</th></tr>
<tr><td>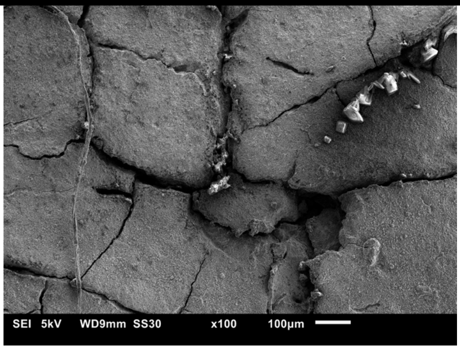
</td><td>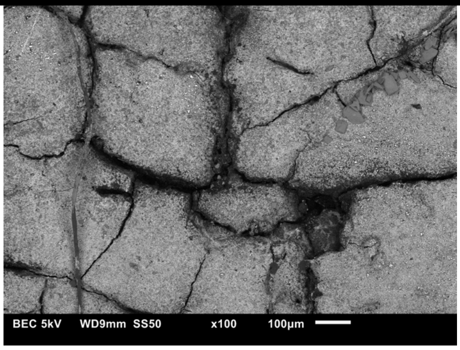
</td></tr>
<tr><td>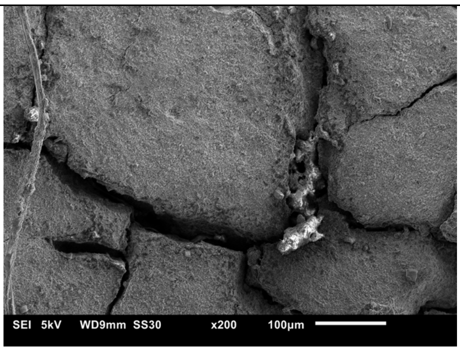
</td><td>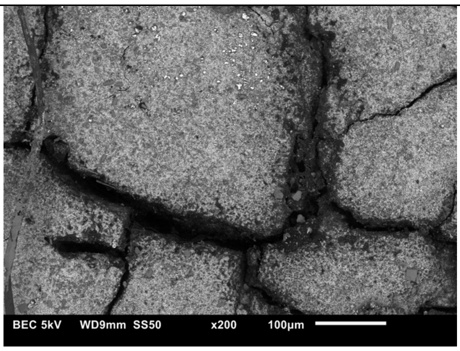
</td></tr>
<tr><td>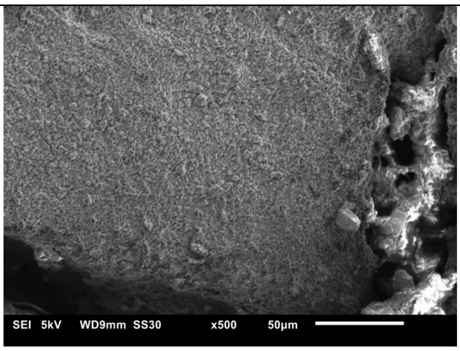
</td><td>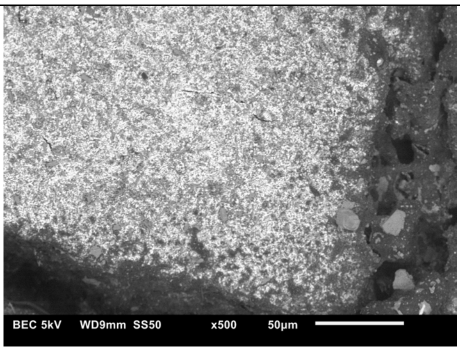
</td></tr>
</table>

We can assume that the carbonate formation on the CL (**Table S8**) has a detrimental effect on the $CO_2R$ performance because it reduces the active area of the CL. It is unclear, however, if the salt formation leads to a slow decrease in $FE_{CO}$ over time or if its effect stabilizes after an equilibration period. During such a period, the salt formation could have reached an equilibrium state, in which the rate of nucleation and growth of crystals equals the rate of dissolution and/or detachment.

**Table S8**: Salt formation on ELAT LT1400W GDE: Scanning electron microscope images of the catalyst layer after the 125 h performance test at different magnifications (x1000 – x5000). **Left**: Secondary electron imaging (SEI) for morphology, **Right**: Back scattered electron composition (BEC) detector for elemental contrast imaging. Dark grey domains in the BEC images indicate the carbon-rich MPL and carbon fibers. Medium grey domains indicate carbonate salt crystals. Light grey domains indicate dispersed Ag nanoparticles in a Nafion matrix.

| **SEI (Morphology)** | **BEC (Composition)** |
|---|---|
| 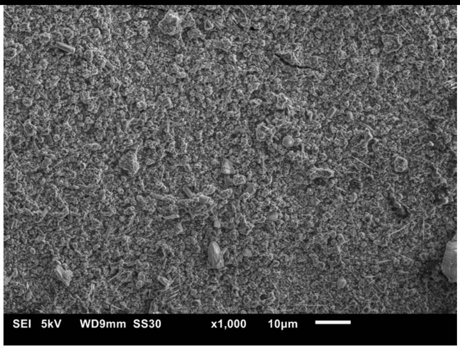  | 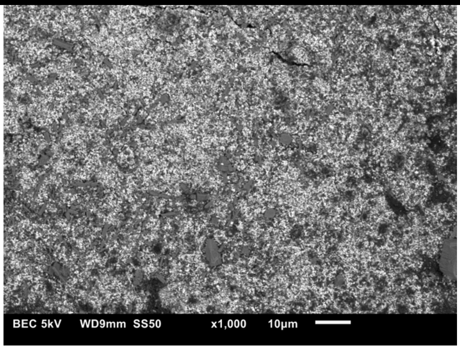  |
| 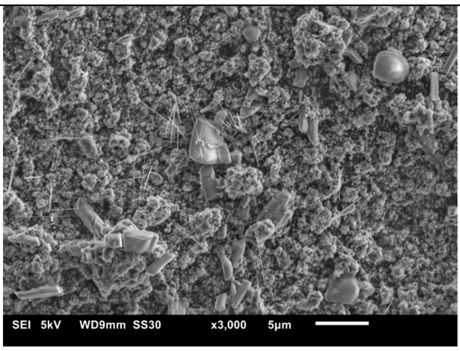  | 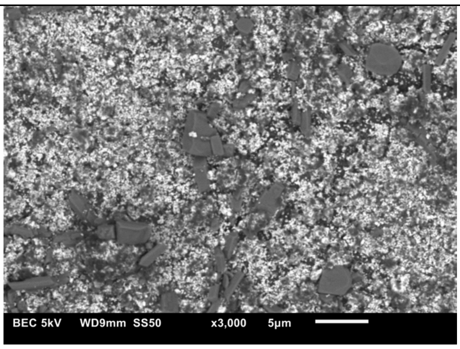  |
| 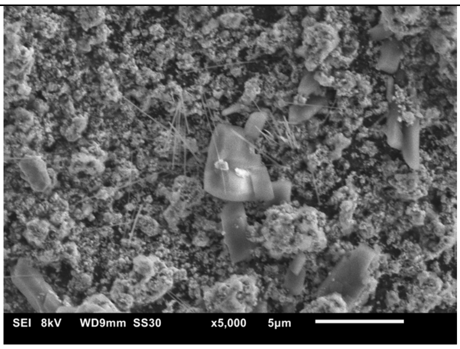  | 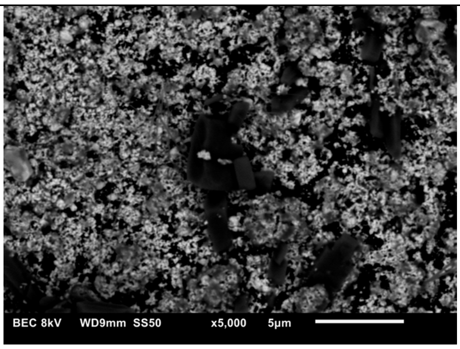  |

We also observed precipitates in the shape of scales on fibers of the CFS (**Table S9**), which have a higher density than the carbon fibers according to the BEC images. These precipitates are probably also carbonate salts.

We measured the static contact angle to assess how much the salt formation affected the wettability. While $\theta_{\mathrm{CFS}}$ did not change significantly after 4.5 h of $CO_2$ electrolysis (**Table S6**), it decreased slightly from 147° (fresh CFS) to 140° after 125 h (**Table S10**). This reduction could be caused by the hygroscopic properties of the salts formed on the carbon fibers. The $\theta_{\mathrm{CL}}$ seems to have fallen from 138° to 78° degrees after the 4.5 h experiment, but then increased again to 102° after the 125 h experiment. The later increase in observed contact angle could be explained by an increase in CL roughness due salt formation and/or mechanical deformation, which can lead to an enhancement of the observed contact angle according to the Cassie-Baxter model.[18]

**Table S9**: Salt formation on ELAT LT1400W GDE: Scanning electron microscope images of the carbon fiber substrate after the 125 h performance test at different magnifications (x100 – x500). **Left**: Secondary electron imaging (SEI) for morphology, **Right**: Back scattered electron composition (BEC) detector for elemental contrast imaging.

| SEI (Morphology) | BEC (Composition) |
|---|---|
| 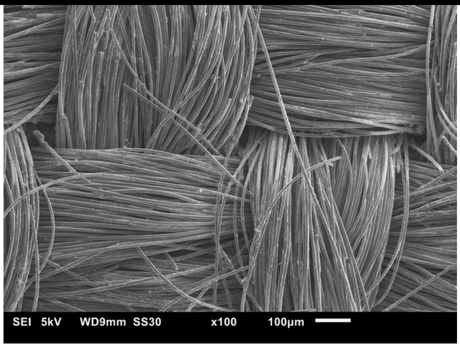 | 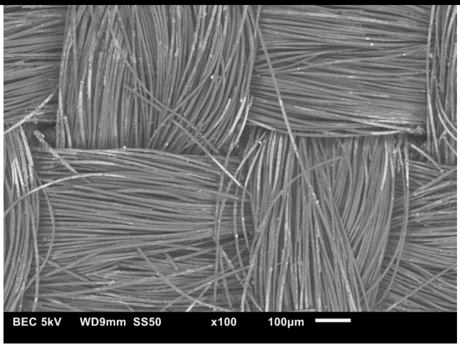 |
| 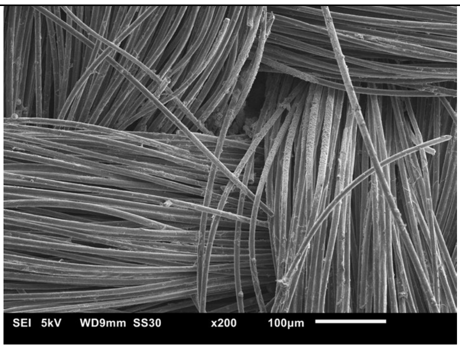 | 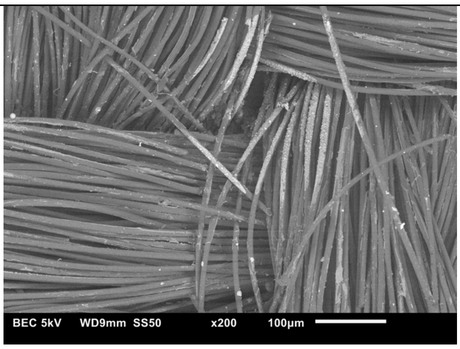 |
| 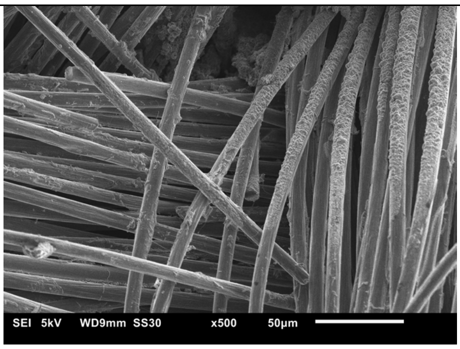 | 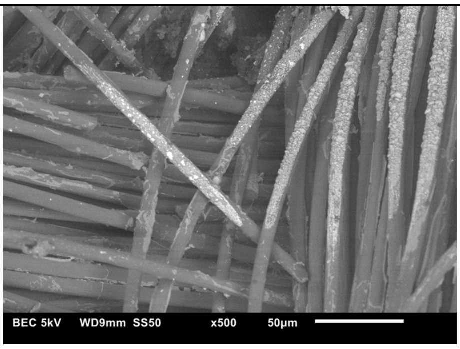 |

**Table S10**: ELAT LT1400W after 125 h performance test: Static contact angles $\theta_i$ ± standard error of different layers $i$.

| Layer | Fresh GDE[1] | After 4.5 h electrolysis (Figure S11)[1] | After 120 h electrolysis (Figure S22) |
|---|---|---|---|
| $\boldsymbol{\theta}_{\mathbf{CFS}}$ | 147 ± 0.8° | 150 ± 1.8° | 140 ± 0.9° |
| $\boldsymbol{\theta}_{\mathbf{MPL}}$ | 150 ± 1.4° | - | - |
| $\boldsymbol{\theta}_{\mathbf{CL}}$ | 138 ± 1.7° | 78 ± 4.1° | 102 ± 3.8° |

The carbonate formation on the CFS (**Table S9**) could also decrease the $FE_{\text{CO}}$ over time (**Figure S22**) because a lower $\theta_{\text{CFS}}$ could lead to a decrease in effective diffusivity of $CO_2$. It is, however, possible that the internal pore surfaces remain unaffected because the precipitation seems to be mostly present on the external surface.

It is difficult to quantify any mechanical deformation because defects were already present in the fresh MPL (**Table S11**). For this reason, it is unclear if the cracks present after 125 h of liquid flow-through were already present before or were created through the mechanical pressure of the liquid.

**Table S11**: Scanning electron microscope images of ELAT LT1400W defects: Comparison of MPL cracks in fresh MPL and CL after 125 h of liquid flow-through.

<table>
<tr><th>Fresh MPL (uncoated)</th><th>CL after 125 h</th></tr>
<tr><td>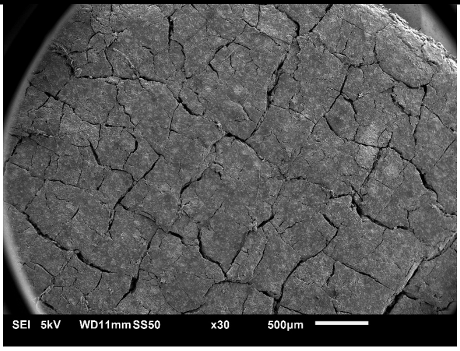
</td><td>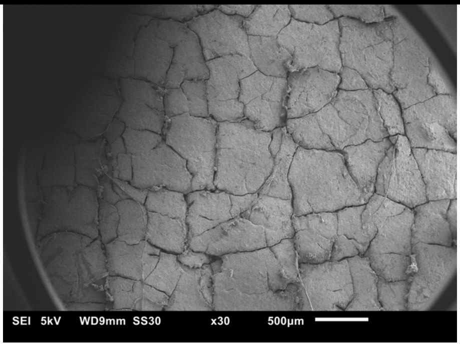
</td></tr>
</table>

The Ag nanoparticles in the catalyst layer were highly stable during the 125 h performance test because they do not seem to have changed in size (**Table S12**). This finding indicates that the Nafion matrix of the CL can effectively prevent aging and/or agglomeration during sustained electrolysis conditions. We complemented the SEM characterization with X-ray diffraction (XRD) measurements. The XRD characterization shows no significant increase in the crystallite size obtained for the peak corresponding to the (111) crystal plane, $L_{(111)}$ , estimated with the Scherrer evaluation (**Figure S24**). This supports the claim that the Ag nanoparticles were highly stable during our experiment.

**Table S12**: BEC images of Ag nanoparticles in Nafion matrix of the catalyst layer (CL): **Left:** Ag nanoparticles in fragments of fresh CL.[1] **Right**: Ag nanoparticles on front face of CL after 125 h performance test.

<table>
<tr><th>Ag nanoparticles in fresh CL</th><th>Ag nanoparticles in CL after 125 h</th></tr>
<tr><td>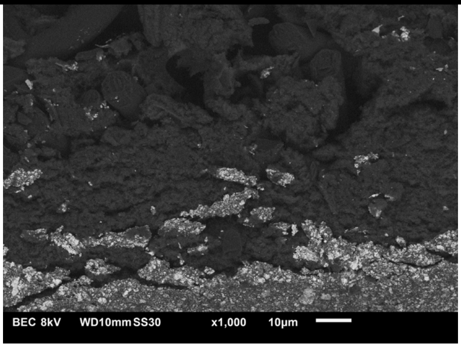
</td><td>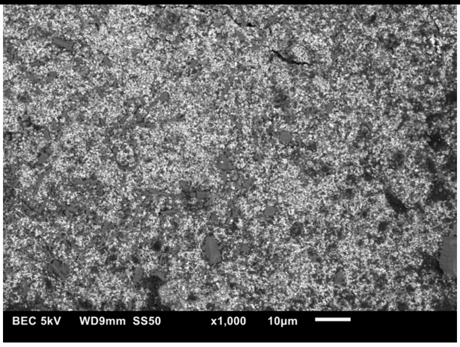
</td></tr>
<tr><td>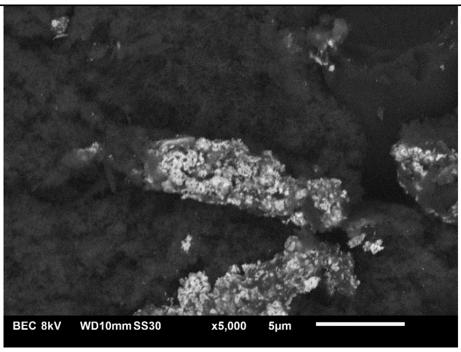
</td><td>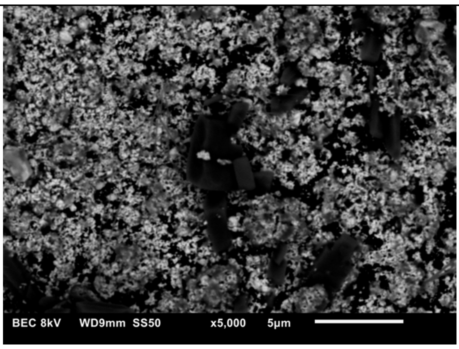
</td></tr>
</table>

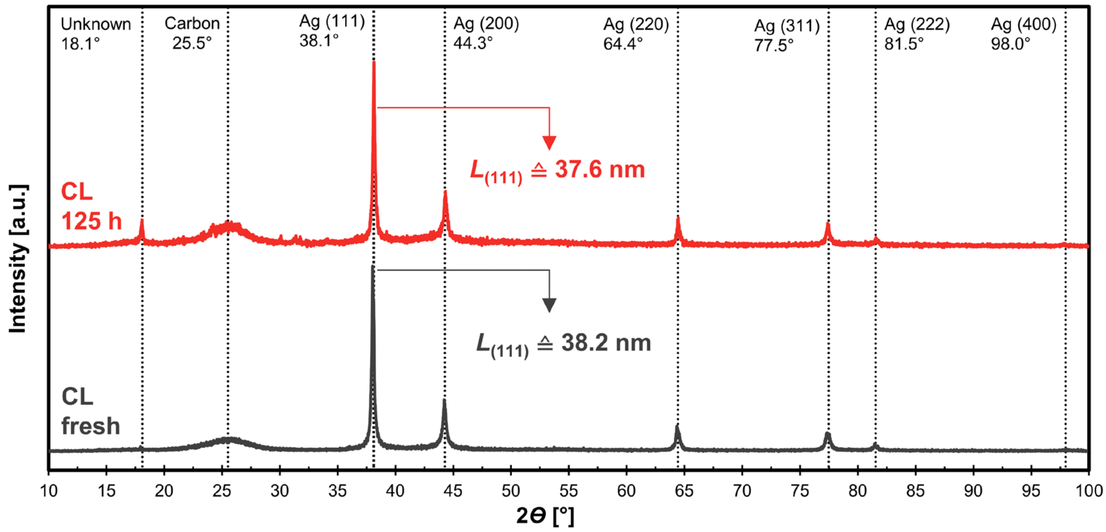


**Figure S24**: X-ray diffractograms for fresh catalyst layer (CL) and for CL after 125 h of electrolysis at 183 mA cm$^{-2}$ obtained with a Bruker D8 Advance diffractometer. X-ray source (40kV, 25 mA): Cu tube with characteristic wavelengths of $\mathrm{K}_{\alpha_1}(100) =$ 1.54060 Å and $\mathrm{K}_{\alpha_2}(100) =$ 1.54439 Å. Position sensitive detector: Lynxeye. The sample was analyzed in the Bragg-Brentano geometry with a step size of 0.01° and an acquisition time of 0.1 s. The sample was illuminated with a divergence slit setting of 5 mm. The diffractograms were processed with DiffracSuite EVA (5.1) software. We corrected the XRD patterns for the $\mathrm{K}_{\alpha_2}$ radiation and carried out a sample height correction. The Powder Diffraction File®(PDF)-2004 database of the International Centre for Diffraction Data powder diffraction file was used for peak assignment. The Scherrer evaluation was used to determine the crystallite size, $L_{(111)}$, corresponding to the (111) plane.

Further, we can assume that there is no significant leaching of Ag into the electrolyte because the concentration of Ag in the electrolyte remained below the detection limit of the ICP-OES analysis (**Table S13**). We detected small concentration of Fe and Ni in the electrolyte. These metals must have leached into the electrolyte from stainless steel parts (Type 316 stainless steel) in the flow path. In theory, these metals could electrodeposit on the cathode and have negative impact on the Faradaic efficiency for $CO_2$R because they catalyze the HER.[5] For this reason electrolyzers should be designed without any stainless steel parts in the flow path to avoid any contamination.

**Table S13**: Leaching and contamination study: Metal concentrations in 1 M $KHCO_3$ electrolyte determined with ICP-OES. Detection limit: 0.01 mg L$^{-1}$.

| Run time | Ag | Fe | Ni |
|---|---|---|---|
| **0 h (Blank)** | < 0.01 mg L$^{-1}$ | 0.02 mg L$^{-1}$ | < 0.01 mg L$^{-1}$ |
| **125 h** | < 0.01 mg L$^{-1}$ | 0.57 mg L$^{-1}$ | 0.26 mg L$^{-1}$ |

## 8.4 Capillary pressure calculation with Owens-Wendt model

We use (S10) to estimate a capillary pressure of $p_C$ = 138 mbar necessary to fill the space within the fiber bundles with electrolyte. It is a modified version of the Young-Laplace equation, which takes the internal contact angle and the heterogeneous GDL surface according to the Owens-Wendt model into account.[19] We approximate the properties of the wetting electrolyte with the properties of pure water. We estimate the radius of the water surface, $r_{H_2O}$, between the individual carbon fibers with 5 µm. The surface tension of water at 20 °C is $\sigma_{H_2O}$ = 0.073 N m$^{-1}$. The dispersive energy component ($\sigma^D_{H_2O}$ = 0.019 J m$^{-2}$) and the overall surface energy ($\gamma_{sub}$ = 0.020 J m$^{-2}$) were taken from Table II from Wood *et al.*.[19] Note that the sign of $p_C$ is negative because the surface tension of water exceeds the term for the attractive forces of the surface. This means that the surface is not wetted spontaneously and is hydrophobic.

$$p_C \approx \frac{4}{r_{H_2O}}\left(\sqrt{\sigma^D_{H_2O}\cdot\gamma_{sub}} - \frac{\sigma_{H_2O}}{2}\right) = -138\ \text{mbar} \qquad (S10)$$

# References


1. Baumgartner, L.M., C.I. Koopman, A. Forner-Cuenca, and D.A. Vermaas, *Narrow Pressure Stability Window of Gas Diffusion Electrodes Limits the Scale-up of CO2 Electrolyzers.* ACS Sustainable Chemistry & Engineering, 2022. **10**(14): p. 4683-4693.
2. Endrődi, B., G. Bencsik, F. Darvas, R. Jones, K. Rajeshwar, and C. Janáky, *Continuous-Flow Electroreduction of Carbon Dioxide.* Progress in Energy and Combustion Science, 2017. **62**: p. 133-154.
3. Duarte, M., B. De Mot, J. Hereijgers, and T. Breugelmans, *Electrochemical Reduction of CO2: Effect of Convective CO2 Supply in Gas Diffusion Electrodes.* ChemElectroChem, 2019. **6**(22): p. 5596-5602.
4. Forner-Cuenca, A., E.E. Penn, A.M. Oliveira, and F.R. Brushett, *Exploring the Role of Electrode Microstructure on the Performance of Non-Aqueous Redox Flow Batteries.* Journal of The Electrochemical Society, 2019. **166**(10): p. A2230.
5. Hori, Y.i., *Electrochemical CO2 Reduction on Metal Electrodes*, in *Modern Aspects of Electrochemistry*. 2008, Springer. p. 89-189.
6. Gostick, J.T., M.A. Ioannidis, M.W. Fowler, and M.D. Pritzker, *Wettability and Capillary Behavior of Fibrous Gas Diffusion Media for Polymer Electrolyte Membrane Fuel Cells.* Journal of Power Sources, 2009. **194**(1): p. 433-444.
7. Weber, A.Z., *Improved Modeling and Understanding of Diffusion-Media Wettability on Polymer-Electrolyte-Fuel-Cell Performance.* Journal of Power Sources, 2010. **195**(16): p. 5292-5304.
8. Mortazavi, M. and K. Tajiri, *Liquid Water Breakthrough Pressure through Gas Diffusion Layer of Proton Exchange Membrane Fuel Cell.* International Journal of Hydrogen Energy, 2014. **39**(17): p. 9409-9419.
9. De Mot, B., J. Hereijgers, M. Duarte, and T. Breugelmans, *Influence of Flow and Pressure Distribution inside a Gas Diffusion Electrode on the Performance of a Flow-by CO2 Electrolyzer.* Chemical Engineering Journal, 2019. **378**: p. 122224.
10. Yang, K., R. Kas, W.A. Smith, and T. Burdyny, *Role of the Carbon-Based Gas Diffusion Layer on Flooding in a Gas Diffusion Electrode Cell for Electrochemical CO2 Reduction.* ACS Energy Letters, 2021. **6**(1): p. 33-40.
11. Zenyuk, I.V., D.Y. Parkinson, G. Hwang, and A.Z. Weber, *Probing Water Distribution in Compressed Fuel-Cell Gas-Diffusion Layers Using X-Ray Computed Tomography.* Electrochemistry Communications, 2015. **53**: p. 24-28.
12. Lamibrac, A., J. Roth, M. Toulec, F. Marone, M. Stampanoni, and F. Büchi, *Characterization of Liquid Water Saturation in Gas Diffusion Layers by X-Ray Tomographic Microscopy.* Journal of The Electrochemical Society, 2016. **163**(3): p. F202-F209.
13. Hatsukade, T., K.P. Kuhl, E.R. Cave, D.N. Abram, and T.F. Jaramillo, *Insights into the Electrocatalytic Reduction of CO2 on Metallic Silver Surfaces.* Physical Chemistry Chemical Physics, 2014. **16**(27): p. 13814-13819.
14. Burdyny, T. and W.A. Smith, *CO2 Reduction on Gas-Diffusion Electrodes and Why Catalytic Performance Must Be Assessed at Commercially-Relevant Conditions.* Energy & Environmental Science, 2019.
15. Moussallem, I., J. Jörissen, U. Kunz, S. Pinnow, and T. Turek, *Chlor-Alkali Electrolysis with Oxygen Depolarized Cathodes: History, Present Status and Future Prospects.* Journal of Applied Electrochemistry, 2008. **38**(9): p. 1177-1194.
16. Nwabara, U.O., E.R. Cofell, S. Verma, E. Negro, and P.J.A. Kenis, *Durable Cathodes and Electrolyzers for the Efficient Aqueous Electrochemical Reduction of CO2.* ChemSusChem, 2020. **13**(5): p. 855-875.

17. Cofell, E.R., U.O. Nwabara, S.S. Bhargava, D.E. Henckel, and P.J.A. Kenis, *Investigation of Electrolyte-Dependent Carbonate Formation on Gas Diffusion Electrodes for $CO_2$ Electrolysis.* ACS Applied Materials & Interfaces, 2021. **13**(13): p. 15132-15142.
18. Quéré, D., *Rough Ideas on Wetting.* Physica A: Statistical Mechanics and its Applications, 2002. **313**(1-2): p. 32-46.
19. Wood, D.L., C. Rulison, and R.L. Borup, *Surface Properties of PEMFC Gas Diffusion Layers.* Journal of The Electrochemical Society, 2010. **157**(2): p. B195.
